# Understanding the impact of radiotherapy fractionation on overall survival in a large head and neck squamous cell carcinoma dataset: a comprehensive approach combining mechanistic and machine learning models

**DOI:** 10.3389/fonc.2024.1422211

**Published:** 2024-08-13

**Authors:** Igor Shuryak, Eric Wang, David J. Brenner

**Affiliations:** Center for Radiological Research, Columbia University Irving Medical Center, New York City, NY, United States

**Keywords:** radiotherapy, head and neck squamous cell carcinoma, causal survival forests, survival, fractionation, biostatistics

## Abstract

**Introduction:**

Treating head and neck squamous cell carcinomas (HNSCC), especially human papillomavirus negative (HPV-) and locally advanced cases, remains difficult. Our previous analyses of radiotherapy-only HNSCC clinical trials data using mechanistically-motivated models of tumor repopulation and killing by radiotherapy predicted that hyperfractionation with twice-daily fractions, or hypofractionation involving increased doses/fraction and reduced treatment durations, both improve tumor control and reduce late normal tissue toxicity, compared with standard protocols using 35×2 Gy. Here we further investigated the validity of these conclusions by analyzing a large modern dataset on 3,346 HNSCC radiotherapy patients from the University Health Network in Toronto, Canada, where 42.5% of patients were also treated with chemotherapy.

**Methods:**

We used a two-step approach that combines mechanistic modeling concepts with state-of-the-art machine learning, beginning with Random Survival Forests (RSF) for an exploratory analysis and followed by Causal Survival Forests (CSF) for a focused causal analysis. The mechanistic concept of biologically effective dose (BED) was implemented for the standard dose-independent (DI) tumor repopulation model, our alternative dose-dependent (DD) repopulation model, and a simple model with no repopulation (BED_simp_). These BED variants were included in the RSF model, along with age, stage, HPV status and other relevant variables, to predict patient overall survival (OS) and cause-specific mortality (deaths from the index cancer, other cancers or other causes).

**Results:**

Model interpretation using Shapley Additive Explanations (SHAP) values and correlation matrices showed that high values of BED_DD_ or BED_DI_, but not BED_simp_, were associated with decreased patient mortality. Targeted causal inference analyses were then performed using CSF to estimate the causal effect of each BED variant on OS. They revealed that high BED_DD_ (>61.8 Gy) or BED_DI_ (>57.6 Gy), but not BED_simp_, increased patient restricted mean survival time (RMST) by 0.5-1.0 years and increased survival probability (SP) by 5-15% several years after treatment. In addition to population-level averages, CSF generated individual-level causal effect estimates for each patient, facilitating personalized medicine.

**Discussion:**

These findings are generally consistent with those of our previous mechanistic modeling, implying the potential benefits of altered radiotherapy fractionation schemes (*e.g.* 25×2.4 Gy, 20×2.75 Gy, 18×3.0 Gy) which increase BED_DD_ and BED_DI_ and counteract tumor repopulation more effectively than standard fractionation. Such regimens may represent potentially useful hypofractionated options for treating HNSCC.

## 1 Introduction

Head and neck squamous cell carcinoma (HNSCC) poses a significant global health challenge, ranking as the seventh most prevalent cancer worldwide. It originates in the mucous membranes of the mouth, nose, and throat (1). HNSCC is classified based on its location, encompassing areas like the oral cavity, oropharynx, nasal cavity, paranasal sinuses, nasopharynx, larynx, and hypopharynx. Depending on the site of origin, it may present as abnormal patches, open sores, bleeding, pain, sinus congestion, sore throat, earache, difficulty swallowing, hoarse voice, breathing difficulties, or lymph node enlargement. HNSCC has the potential to metastasize to other parts of the body, contributing to over 800,000 new cancer diagnoses annually (2, 3).

Radiotherapy is integral to HNSCC treatment. The conventional approach involves administering 2 Gy doses daily on weekdays, accumulating to a total of 70 Gy, with a focus on the primary tumor and affected lymph nodes. Recent decades have seen significant technological advancements, especially with the adoption of intensity-modulated radiotherapy (IMRT). This innovation enables precise delivery of high-dose radiation to the tumor, minimizing exposure to surrounding healthy tissues. The evolution in technology significantly reduces both immediate and delayed toxicity associated with the treatment.

However, treating HNSCC with radiotherapy is still challenging due to several factors (4–7). The intricate anatomy of the head and neck, coupled with its proximity to critical structures, poses challenges in delivering high doses of radiation without significant side effects. Radiation-induced side effects can substantially impact a patient’s quality of life, and adherence to conventional fractionation schedules can be challenging, particularly for those with limited resources. Factors such as comorbidities, substance use, and underinsurance further complicate the treatment landscape. Timely completion is crucial, but obstacles like transportation, lack of caregiver support, and financial strain may hinder adherence. The aging HNSCC population faces additional challenges, given their increased susceptibility to severe toxicity. Addressing these challenges could offer benefits by reducing the burden and duration of radiation therapy, making it more logistically feasible without compromising efficacy.

HNSCC tumors testing negative for Human Papillomavirus (HPV) can demonstrate rapid growth and resistance to radiation therapy. HPV is a group of more than 200 related viruses, with several types linked to cancer development, particularly in the genital and oropharyngeal regions (8). HPV-negative cancers, often associated with tobacco and alcohol use, tend to be more aggressive and less responsive to treatment, with recurrence rates exceeding 35% for advanced stages (9). Despite treatment advancements, cancer recurrence remains a major issue, with a locoregional recurrence rate of 15–50%. While salvage surgery may offer a curative option for patients with resectable locoregional recurrence, it is often not feasible or only possible with severe complications and limited success rates. Furthermore, advanced and recurrent cancers often develop resistance to treatment, making them more challenging to manage (10).

These factors necessitate ongoing research for more effective, less toxic, and logistically simplified HNSCC radiotherapy strategies (11). Radiobiological modeling is important for achieving these goals (12). The clinical utility of the linear-quadratic (LQ) model lies in its ability to compare fractionation schedules and predict radiation responses. In this model, tumor sensitivity to dose per fraction is governed by the α/β ratio. For HNSCC the α/β ratio is relatively high (around 10 Gy), suggesting that its sensitivity to large doses per fraction is not as large as for some cancers (e.g. breast and prostate) with smaller α/β ratios. For this reason, hypofractionation involving doses per fraction >2 Gy was not as thoroughly investigated for HNSCC as for these other cancers, and was mainly used with palliative rather than curative intent for HNSCC. Instead, many HNSCC clinical trials have investigated the opposite approach of hyperfractionation with smaller fractions twice or three times daily to exploit the radiobiological distinction between tumor and normal tissue (13). The administration of small fractions twice per day decreases the incidence of late toxicity, enabling the delivery of higher total radiation doses compared to conventional dosing. Another alternative - accelerated radiotherapy - addresses tumor repopulation concerns by delivering doses of 1.8–2 Gy twice daily or more than five fractions per week, thereby reducing the overall treatment time (12). The reduction in overall treatment time serves to mitigate tumor repopulation. Both strategies have the potential to enhance tumor control.

To address these challenges comprehensively, we adopt a sequential machine learning approach. Initially, we employ Random Survival Forests (RSF) to broadly analyze survival data and identify significant predictors of outcomes. This is followed by a targeted causal investigation using Causal Survival Forests (CSF), which allows us to delve deeper into the causal relationships between treatment variables and patient survival. This pipeline approach ensures a thorough exploration of the data, starting with general pattern identification and leading to specific causal inferences.

Several randomized trials have investigated various radiotherapy schedules for head and neck cancer, but conflicting results regarding tumor control and survival have emerged. The inconsistency is mainly attributed to trial heterogeneity and small sample sizes. Despite these challenges, the trials suggest that modifications in fractionation are often linked to more frequent acute side effects, while late toxicity rates are similar or less frequent compared to conventional fractionation radiotherapy.

The Meta-Analysis of Radiotherapy in Carcinomas of Head and Neck (MARCH) revealed that altered fractionation radiotherapy is associated with improved overall survival and progression-free survival when compared to conventional fractionation radiotherapy (13). The analyzed trials were categorized based on specific altered fractionation techniques. These categories included hyperfractionation, involving a higher total dose administered through twice-daily fractions within the same overall treatment time; moderate acceleration, maintaining an unchanged total dose but delivered more expeditiously (typically about 1 week faster); and very accelerated radiotherapy with dose reduction, reducing the duration by 50% or more, accompanied by a total dose decrease of approximately 15%. The meta-analysis notably excluded trials investigating hypofractionated radiotherapy, primarily used in palliative cases with doses per fraction >2.5 Gy. This comprehensive analysis affirmed the effectiveness of altered fractionation radiotherapy, especially hyperfractionation, over conventional fractionation radiotherapy.

However, hyperfractionation imposes a logistical challenge which can result in reduced compliance with the therapy. Hypofractionation using fewer larger fractions delivered over a shorter overall time alleviates this issue, and the shortened treatment time is useful to counteract repopulation of quickly growing HNSCC tumors. Navigating the radiobiological complexities of hypofractionation, where the delicate balance between tumor control and toxicity is crucial, represents a key consideration (7). The adoption of accelerated regimens hinges on mucosal tolerance, with hypofractionation demonstrating feasibility when considering mucosal tolerance, resulting in shorter acute side effects and improved tolerability. Initiatives transitioning from conventional to hypofractionation must carefully weigh the impact on late toxicity. While shorter regimens may mitigate late effects, the heightened radiosensitivity of tissues during prolonged radiation courses poses challenges. Studies comparing 3 and 5 weeks of radiation therapy for HNSCC show varied outcomes in tumor control and late toxicity. Successful implementation of hypofractionation in HNSCC treatment requires a comprehensive approach incorporating modern techniques, systemic therapy, and vigilant monitoring.

Importantly, there has been little progress for HPV-negative HNSCCs in the past two decades (7). Despite efforts to explore systemic therapies beyond cisplatin, achieving better disease control remains challenging. However, potential advancements are anticipated with emerging agents and adjuvant therapies. Hypofractionation, endorsed through noninferiority trials across various cancers, has become a preferred practice in multiple disease sites, enhancing treatment efficiency and reducing costs. The ongoing investigation into ultrahypofractionation (≤5 fractions) represents a second wave of studies aiming to further optimize treatment approaches.

Amidst ongoing HNSCC clinical trials, such as HEADLIGHT (Mayo Clinic), NCT05075980; NCT04284540 (Mount Sinai); HYPORT (NCT04403620), HYHOPE (NCT04580446) at the University of Texas Southwestern; and DEHART (NCT04477759), HART-HN (NCT 05120947), HyPR-HN (NCT05538533) at the Medical College of Wisconsin, there is a discernible interest in adopting hypofractionation for head and neck cancer. While hypofractionation is already applied in specific cases, such as early-stage glottic cancer, stereotactic body radiation therapy for reirradiation, palliation, and heavy ion therapy, it is not universally applicable for all head and neck cancers. Existing uncertainties, such as the absence of late toxicity assessments beyond 5 years in ongoing studies and limited randomized comparisons with conventional fractionation, underscore the need for a cautious approach. Some trials employ a time-to-event continuous reassessment methodology (TiTE-CRM), an adaptive statistical approach, to assess delayed toxicity. Key considerations include the safety of delivering radiosensitizing cisplatin with hypofractionation, economic implications, and the generalizability of findings beyond specialty centers. The ongoing exploration of conformal hypofractionation should prompt a comprehensive evaluation of its potential benefits for patients with HNSCCs.

Our previous analyses (14, 15) of classic radiotherapy-only HNSCC clinical trials data using mechanistically-motivated quantitative models of tumor repopulation and killing by radiotherapy predicted that hypofractionation involving increased doses/fraction and reduced overall treatment durations, or hyperfractionation with twice-daily fractions, improve tumor control and reduces late normal tissue toxicity, compared with protocols using 35x2 Gy fractions spread over 7 weeks. Specifically, in the first study (16) we explored an alternative dose-dependent (DD) model of accelerated tumor repopulation (AR), where the onset time and rate of AR depend on the number of tumor clonogens killed, thus on radiation dose and dose-fractionation. This model produced better fits to a wide range of clinical data from HNSCC clinical trials, compared to the standard dose-independent (DI) repopulation model, where the onset time and rate of AR do not depend on radiotherapy details. This model, which assumes that repopulation occurs when a threshold survival fraction is reached, is detailed in the paper by Shuryak et al. (2018) (14). In the second study (17), we performed systematic radiobiological optimization using both DD and DI repopulation models to identify fractionation schemes that improve the balance between tumor control probability (TCP) and long-term normal tissue complication probability (LNTCP).

This research suggested that both hypofractionated schedules with doses >2 Gy/fraction, and twice-daily treatments with <2 Gy/fraction, with reduced overall treatment times, can substantially increase TCP while decreasing LNTCP, compared to a standard 35x2 Gy protocol. This general conclusion applied to both the DI and DD models, although the numerical predictions differed somewhat between models. Hypofractionation and twice-daily hyperfractionation are related in their radiobiological effects because both approaches increase the “intensity” of tumor cell killing per day, and this pattern applies regardless of the details of the repopulation model. Consequently, hypofractionation or its close variant, accelerated hyperfractionation, prove to be efficient strategies in overcoming tumor repopulation, especially in fast-growing tumors like HNSCC.

The main limitations of these modeling studies were: (1) Only classic radiotherapy-alone (no chemotherapy) HNSCC clinical trials were analyzed because the DD and DI models were not developed to handle chemotherapy effects. The treatment approaches and technology implemented in those trials are by now largely outdated. (2) The data were available only in summary form: group averages for different arms of the clinical trials, instead of individual-level patient data. (3) Some important clinical variables such as HPV status were not available.

The objective of the current paper is to scrutinize the validity of the main conclusions of these previous modeling studies about the usefulness of altered radiotherapy fractionation through the lens of contemporary HNSCC patient treatment data, evaluating patients who have undergone modern treatment techniques. To achieve this, we used a comprehensive dataset from the RADCURE project, encompassing records of 3,346 patients treated for head and neck cancer at the University Health Network in Toronto, Canada, from 2005 to 2017 (18, 19). This dataset is particularly valuable due to its extensive coverage of relevant parameters, including radiotherapy dose and fractionation, chemotherapy, various clinical variables, and patient survival outcomes.

We used a two-step analysis approach on the RADCURE data set, which combines mechanistic modeling concepts with state-of-the-art machine learning techniques, beginning with Random Survival Forests (RSF) for an exploratory analysis and followed by Causal Survival Forests (CSF) for a focused causal analysis. This approach is informed by previous work where RSF and Shapley Additive Explanations were effectively employed to model nonlinear relationships in radiation exposure studies. This study aims to thoroughly investigate the effects of radiotherapy fractionation on overall patient survival (OS) and cause-specific mortality (deaths from the index cancer, other cancers or other non-cancer causes), contributing valuable insights to the ongoing discourse on optimizing head and neck cancer treatment strategies.

Importantly, conventional statistical and machine learning approaches primarily emphasize prediction based on variable correlations (20). These methods produce associations rather than causation regarding the influence of individual features (*e.g.*, radiation dose). In contrast, Causal Machine Learning (CML), such as causal survival forests (CSF), can be used for establishing causal relationships between variables, facilitating the generalization of results across various scenarios and enabling targeted interventions (20–23).

Predictive and causal modeling approaches are fundamentally distinct (20, 24–26). In predictive tasks, the objective is to accurately predict an outcome variable, such as patient survival time, based on predictor variables (features). The accuracy of these predictions for new datasets depends on the resemblance of data distributions and correlation structures to the initial training data. In contrast, causal analysis involves modeling how changes in a specific feature, such as radiation exposure, influence the outcome. Causal modeling’s utility extends beyond the distributions of training data, making it adaptable to various datasets, even with disparate variable correlations. This distinction highlights that predictive modeling is an unreliable approach for causal inference, especially in observational data. Predictive models tackle associations, while causal models explore interventions and counterfactuals, assessing the effects of modifying causal variables (20). Maximizing prediction accuracy during training may introduce biases, such as collider bias and overcontrol bias, which can potentially lead to errors in causal inference (27–30). Common techniques used in predictive modeling, like regularization and feature selection, may also yield inaccurate causal inferences. Predictive models can only be causally interpreted when the feature of interest is independent not only of other features but also of unobserved confounders.

In contrast, CML methods are explicitly designed to model cause-and-effect relationships, even within complex data featuring confounders. CML is not constrained by strict assumptions about the shapes of causal relationships. Its main assumptions include the stability of cause-and-effect relationships, the absence of reverse causation, ignorability, and positivity. The ignorability assumption posits the absence of unmeasured confounding variables affecting both the treatment variable (the focal variable for causal investigation) and the outcome variable (the variable under causal scrutiny). Essentially, it presumes that all relevant confounding variables are both measured and considered in the analysis. While ignorability is not directly assessable, refutation methods can indirectly evaluate it. Positivity assumes that the likelihood of receiving a particular treatment level (exposure) exceeds zero for all strata of covariates (confounding variables). This implies that every subgroup within the population has a nonzero chance of being exposed to the treatment of interest. Failing to meet the positivity assumption can make estimating causal effects challenging, as there may be groups where the treatment is never observed, rendering it impossible to assess its impact within those groups.

Another very important capability of several CML methods is the doubly robust estimator property. A doubly robust estimator (DRE) remains unbiased even if either the treatment or outcome model is correctly specified, but not necessarily both. This flexibility enhances the robustness of causal inferences, as it allows for some degree of model misspecification without compromising the reliability of the estimated treatment effects. For example, suppose we are investigating the causal effect of a specific radiotherapy protocol on the survival time of cancer patients. In this scenario, there will be two models: (1) The *treatment model*, which estimates the effect of radiotherapy on survival time and accounts for various factors, including dose, duration, age, stage of cancer, comorbidities and other patient characteristics. (2) The *outcome model*, which predicts patient survival time and considers the same relevant variables as the first model. The DRE property implies that even if only one of these two models (but not both) is correctly specified, the estimated causal effect of the radiotherapy remains unbiased. This robustness is valuable because, in practice, it is very challenging to perfectly specify both models due to the complexity of medical scenarios and the presence of unobserved variables. Thus, the DRE property ensures that causal inferences remain reliable and unbiased even when there is some degree of uncertainty or misspecification in the models used to estimate treatment effects and predict outcomes.

While CML methods have been gaining recognition in a range of disciplines (22, 23, 31, 32), their adoption in radiation oncology so far remains limited. CML offers a more thorough and accurate comprehension of these relationships, with the capacity to estimate effects at both population and individual levels, holding substantial potential for informing radiation oncology decision-making.

In this study, we combined the strengths of three types of radiobiological modeling joined into a coherent analysis pipeline: (1) The mechanistic concept of biologically effective dose (BED) was implemented for the standard dose-independent (DI) tumor repopulation model, for our alternative dose-dependent (DD) repopulation model, and for a simple model with no repopulation (BED_simp_). (2) A powerful predictive machine learning algorithm - random survival forests (RSF) – was employed as an initial step to model patient overall survival (OS) and cause-specific mortality (deaths from the index cancer, other cancers or other non-cancer causes) using the BED variants together with clinical variables. (3) Targeted causal inference analyses were then performed using the CSF algorithm to estimate the causal effect of each BED variant separately on OS. We believe that the results of these analyses deepen the current insights into how radiotherapy fractionation affects the effectiveness of HNSCC treatment.

## 2 Materials and methods

A detailed description of the data processing and machine learning analyses is provided in **Supplementary Methods** in the **Supplementary Materials**. The main aspects are provided below.

### 2.1 Data collection

The dataset was obtained from the RADCURE project, consisting of 3,346 head and neck cancer patients treated with definitive radiotherapy (RT) at the University Health Network in Toronto, Canada (18, 19). This dataset is very useful for investigating the effects of different radiotherapy fractionation schemes on HNSCC patient survival because it includes a variety of total doses and doses per fraction. To provide a comprehensive overview of the treatment regimens used in the dataset, we analyzed the distribution of different fractionation schedules. **Table 1** summarizes the number of patients treated with various numbers of fractions and doses per fraction. It shows that while most patients were (expectedly) treated with the standard 35 fraction/70 Gy regimen, 4 other regimens were represented by >50 patients each, indicating the diversity of the RADCURE data set.

**Table 1:** Detailed breakdown of radiotherapy fractionation schedules in the RADCURE data set.

Number of Fractions	Total dose (Gy)	Number of Patients
35	70.00	2289
25	60.00	386
40	64.00	263
20	51.00	227
33	66.00	91
30	60.00	28
25	50.00	19
20	50.00	9
34	68.00	6
32	64.00	4
40	60.00	3
31	62.00	2
33	69.96	2
60	66.00	2
20	52.00	1
20	55.00	1
21	53.55	1
22	52.80	1
27	60.00	1
30	66.00	1
33	59.40	1
34	70.00	1
36	54.40	1
37	69.60	1
39	74.00	1
40	62.40	1
40	50.80	1
41	67.60	1
45	66.00	1

### 2.2 Data preprocessing and feature selection

The RADCURE data set contained numerous clinical variables, and for analysis we selected the following most relevant ones, trying to avoid redundancy: Age (patient age, years), Sex (0=female, 1=male), Smoking_PY_ (number of packs smoked in a year), Stage_numeric_ (AJCC 7th edition staging categories, converted into integers of 0–4), HPV (tumor HPV status determined by p16 IHC ;+/- HPV DNA by PCR), Chemo (1=received concurrent chemoradiotherapy, 0=did not receive concurrent chemoradiotherapy), RT_year_ (calendar year of the radiotherapy treatment), Status (binary indicator of vital status at last contact date), Length_FU_ (duration of follow up from diagnosis to last contact date in years). The HPV status was categorical, indicating positive, negative, or unknown (missing). To incorporate this information into our analysis, we applied one-hot encoding, representing HPV- as the default, while creating separate binary columns for HPV_Positive_ and HPV_Unknown_. The Status and Length_FU_ variables were the outcome variables, indicating overall survival (OS). The data were imported and analyzed using the *R* and Python programming languages.

In addition to studying OS, we performed a targeted analysis of cause-specific mortality in HNSCC patients, distinguishing deaths from the index cancer, other cancers or other (non-cancer) causes. In this analysis we also assessed the contributions of head and neck cancer diagnosis site to each cause of death. We selected only those sites which contained data for ≥20 patients each (Esophagus, Hypopharynx, Larynx, Lip Oral Cavity, Nasal Cavity, Oropharynx), and discarded sites with smaller numbers of patients and entries with unknown diagnosis site. This data subset contained 2,651 patients. These selection criteria were designed to exclude those diagnosis sites for which too few samples were available to be informative. The different diagnoses sites were coded as separate columns of binary (0 or 1) variables, with 1 indicating that the given sample had the tumor at the given site.

### 2.3 Calculation of biologically effective dose for different tumor killing and repopulation models

To compare the effects of various radiotherapy regimens present in the RADCURE data set, we employed the well-established Biologically Effective Dose (BED) concept (33). If tumor repopulation is neglected, a simplistic BED can be calculated as follows, where m is the number of fractions, d is dose per fraction, and r is the α/β ratio (assumed to be 10 Gy for HNSCC):


$$BED_{simp}=m\;d\;\left(1+\frac{d}{r}\right)$$

A more advanced BED version which includes accelerated tumor repopulation (AR) which is assumed to begin at a fixed onset time T_k_ is based on the work of Withers et al (34). Since T_k_ is assumed to be independent of radiotherapy details such as dose or dose/fraction, we called this the “dose independent” (DI) model, and the consequent BED is labeled BED_DI_. Based on our previous publication (14), the equation for BED_DI_ is as follows, where λ = accelerated tumor repopulation rate, g = background slow repopulation rate (not accelerated), T_k_ = onset time for accelerated repopulation, T = total radiotherapy treatment time:


$${\text{BED}}_{\text{DI}}=\left[\text{m α d}\frac{\text{d }+\text{ r}}{\text{r}}-\text{ g T }-\text{ λ max}\left(0,\text{ T }-{\text{ T}}_{\text{k}}\right)\right]/\text{α}$$

As another alternative, we considered our proposed “dose dependent” (DD) tumor repopulation model, where both the onset time and rate of AR are assumed to depend on the average fraction of tumor cells killed by radiotherapy each day (14). In other words, in this model the tumor responds to a higher “intensity” of tumor cell killing by radiotherapy by starting AR earlier and increasing its rate. AR is assumed to begin when the natural logarithm of the tumor cell surviving fraction drops below a certain value –C. The equation describing BED_DD_ from this model is below:


$${\text{BED}}_{\text{DD}}=\frac{\left[\text{r λ}\left(\text{exp}\left(-\text{m α d}\frac{\text{d }+\text{ r}}{\text{T r}}\right)-\;1\right)\max\left(0,\;-\text{T}\frac{-\text{m α d }\left(\text{d }+\text{ r}\right)+\text{ C r}}{\text{m α d }\left(\text{d }+\text{ r}\right)}\right)+\;\left(\text{α d m }-\text{ T g}\right)\text{ r }+\text{ m α }d^{2}\right]}{\text{r α}}$$

Details of the derivations of BED_DI_ and BED_DD_ are described in the **Supplementary Methods**. The parameters were taken from reference (14). They were as follows: BED_DI_: α = 0.069 Gy^-1^, λ = 0.035 days^-1^, T_k_ = 28.6 days; BED_DD_: α = 0.224 Gy^-1^, λ = 1.17 days^-1^, C = 14.5. The default α/β ratio r was 10 Gy for both models (14). The resulting BED_simp_, BED_DI_ and BED_DD_ variables were included in the RADCURE data set as predictors of OS. These parameter values were derived from fitting the DD and DI models to a collection of classical HNSCC clinical trials involving different radiotherapy fractionation schemes.

Since it is known that the α/β ratio for HNSCC can vary considerably among different studies and data sets (35), in the subset analysis described above for cause-specific mortality we also assessed sensitivity of model predictions to this parameter by including in the model different versions of BED_simp_, BED_DI_ and BED_DD_ which differed from each other by using different α/β values, 7, 10 or 13 Gy. These variables were labeled with extra subscripts, *e.g.* BED_DD 7_, BED_DD 10_ and BED_DD 13_.

### 2.4 Machine learning pipeline for predictive and causal analysis

Our study utilizes a unified two-step machine learning pipeline to analyze the effects of radiotherapy fractionation on overall survival. In the first step, we employ Random Survival Forests (RSF) to conduct a general analysis of the dataset, leveraging RSF’s capability to handle censored data and capture complex interactions between features. This exploratory phase helps in identifying significant predictors of patient outcomes and sets the stage for a more detailed investigation. The second step involves deploying Causal Survival Forests (CSF) to specifically explore the causal impact of identified variables on survival. This targeted approach allows us to substantiate the patterns observed in the RSF analysis with a causal perspective, providing insights into how changes in treatment variables could potentially affect patient survival. This sequential utilization of RSF and CSF underscores our comprehensive strategy to not only predict survival outcomes but to understand the underlying causal mechanisms. In each analysis, the data set was randomly divided (70:30) into training and testing portions, with all model fitting and optimization being performed on the training portion and the testing portion being withheld for evaluation. The two steps of this approach are described below.

*Step 1: Predictive Machine Learning Analysis using Random Survival Forests (RSF).*

We utilized Random Survival Forest (RSF), a predictive machine learning method, to model OS using all the other available variables (features) in the data set. The goals of this analysis were to identify: (1) How accurately can OS be predicted using the available variables? (2) Which variables are most important contributors to these predictions? (3) How does radiotherapy, represented by the BED_DD_, BED_DI_ and BED_simp_ variables, contribute to predicting OS or cause-specific mortality? Separate RSF analyses were performed for all patients in the data set, and separately for the HPV- patients only. SHapley Additive exPlanations (SHAP) values are a state of the art method for interpreting complex machine learning models, such as those used here. We calculated SHAP values for all features in the RSF model and visualized them. These calculations were implemented using the *scikit-survival* Python library.

As mentioned above, we also conducted a subset analysis focused on cause-specific mortality, distinguishing deaths from the index cancer, other cancers or other (non-cancer) causes, and evaluating the contribution of diagnosis site and α/β ratio. Since multiple competing causes of death were modeled in this scenario, we used the RSF variant for competing risks, implemented in the *randomForestSRC R* package (36). The RSF model handles competing risks by simultaneously considering multiple mortality causes. At each node, the model uses a modified log-rank test to choose splits that best separate causes of death. Each terminal node represents data subsets with similar characteristics and predicted outcomes. These competing risk modeling results were visualized using the Cause-Specific Cumulative Hazard Function (CSCHF) and Cumulative Incidence Function (CIF) plots. The CSCHF illustrates the rate of death from a specific event type while considering the presence of other competing events. In contrast, the CIF estimates the marginal probability of death based on its cause-specific probability and overall survival probability. These metrics are vital for comprehending the survival experience involving multiple competing events and offer more interpretable estimates than traditional survival analysis methods. Variable Importance (VIMP) scores, which assess the significance of each feature in predicting outcomes (different mortality causes), were also generated. VIMP is determined by the increase in prediction error when the feature’s values are randomly permuted. A larger increase in prediction error indicates greater importance of the feature. High positive VIMP values signify strong predictive importance.

*Step 2: Causal Machine Learning Analysis Using Causal Survival Forest (CSF).*

The CSF algorithm, implemented using the causal_survival_forest function of the *grf R* package (23, 37), was employed to quantify the *causal* effects of each BED variant (BED_DD_, BED_DI_ or BED_simp_) on OS. The CSF algorithm uses a binary (0 or 1) causal/treatment variable as the input. Consequently, we converted continuous BED_DD_, BED_DI_ or BED_simp_ into binary variables by using manually defined cut-points guided by the SHAP analysis results from the RSF model described above. For example, the examination of RSF-generated SHAP values for BED_DD_ suggested a nonlinear response, where a clear reduction in patient mortality was associated with BED_DD_ values >61.8 Gy. Consequently, a causal variable for CSF analysis was created where BED_DD_ values ≤61.8 Gy were mapped to 0, and BED_DD_ values >61.8 Gy were mapped to 1. The same approach was used to binarize BED_DI_ and BED_simp_, but with different cutoff values.

Consequently, three separate CSF analyses were performed, using binarized versions of either BED_DD_, BED_DI_ or BED_simp_ as the causal/treatment variable. In each case, the other two BED versions were not included in the data set. For example, if binarized BED_DD_ was the causal variable, BED_DI_ and BED_simp_ were not included. The set of covariates/potential confounders was the same in each analysis: Sex, Smoking_PY_, Stage_numeric_, HPV_Positive_, HPV_Unknown_, Chemo, RT_year_, Age.

Two estimands (metrics) were selected to describe the causal effect of each BED variant. They were RMST (Restricted Mean Survival Time) and survival probability (SP). RMST represents the average survival time up to a specific time point (e.g., a fixed follow-up time or a certain event occurrence). Notably, it offers a straightforward and easily communicable representation of the average survival duration up to a specified time point. Its significance is particularly pronounced in clinical research, as it provides a clinically comprehensible summary of time-to-event data, facilitating the evaluation of intervention effectiveness and therapeutic outcomes. Additionally, RMST exhibits robustness in the face of violations of the proportional hazards assumption, rendering it well-suited for diverse study scenarios where alternative methods may prove less effective. In comparison, SP refers to the likelihood of an event (death in this case) occurring beyond a given time point. In causal survival forests, SP provides insights into the probability of survival (or event-free survival) at specific time intervals. Both RMST and SP were calculated for various times after treatment. Various sensitivity analyses and refutation tests, described in more detail in **Supplementary Methods**, were performed to evaluate the robustness of CSF results.

## 3 Results

### 3.1 Predictive RSF analysis

**Figure 1:**
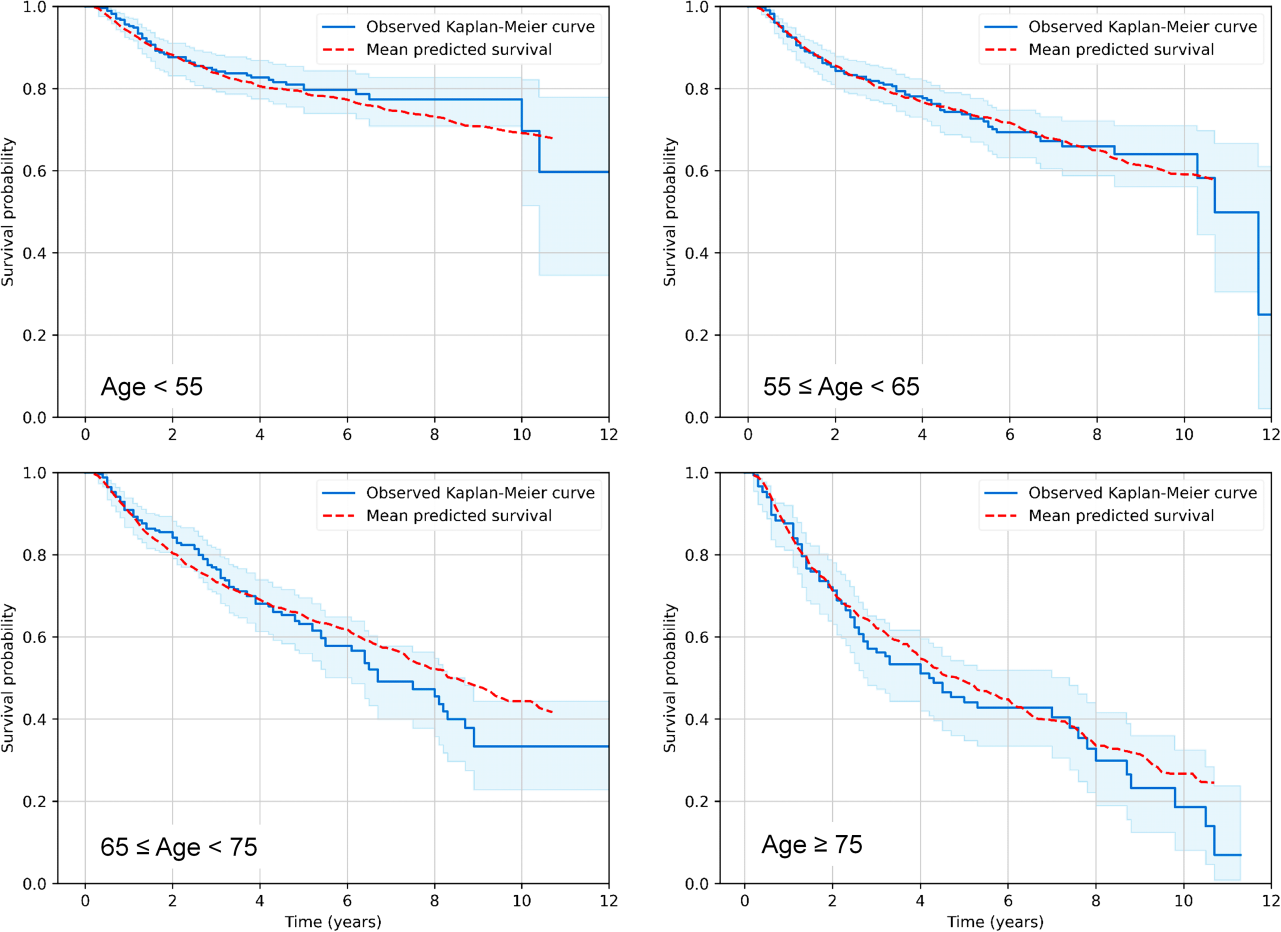
Visualization of predictive performance of the RSF model for OS on testing data. Mean predicted Kaplan-Meier survival curves for patients in different age ranges (red dashed curves) are compared with observed patterns (blue solid curves), as function of time after treatment. Blue shaded regions represent 95% confidence intervals for observed survival.

In the first step of our analysis pipeline, we utilized RSF to explore and predict overall survival (OS) or cause-specific mortality (distinguishing deaths from the index cancer, other cancers or other non-cancer causes) in the RADCCURE patients using the BED variants and clinical variables as features (predictors). Visually, RSF predictions for OS agreed quite well with the observed data: a comparison of mean RSF-predicted and observed Kaplan-Meier survival curves for patients in different age ranges is shown in **Figure 1**. As expected, OS was reduced for patients of older ages compared with those in younger age groups, and the RSF model captured these patterns adequately. The effects of all other variables were lumped together within each age group, so the plots in **Figure 1** are intended only as an overall visualization of model agreement with the data.

Quantitatively, predictive performance of the RSF algorithm with tuned hyperparameters (min_samples_leaf = 30, min_samples_split = 2, n_estimators = 52) was assessed on training data using 10-fold cross validation (CV), and separately on testing data. On training data (70% of the data set), it generated a mean concordance score (c-index) of 0.759 on training folds and 0.730 on testing folds. On separate testing data (30% of the data set) this model had a c-index of 0.718. Very similar performance was achieved if only BED_DD_, rather than all three BED variants (BED_DD_, BED_DI_ and BED_simp_) were included in the model. These results suggest that RSF performance was good, and the model was not overfitting the training data much and was able to generalize well to testing data.

**Figure 2:**
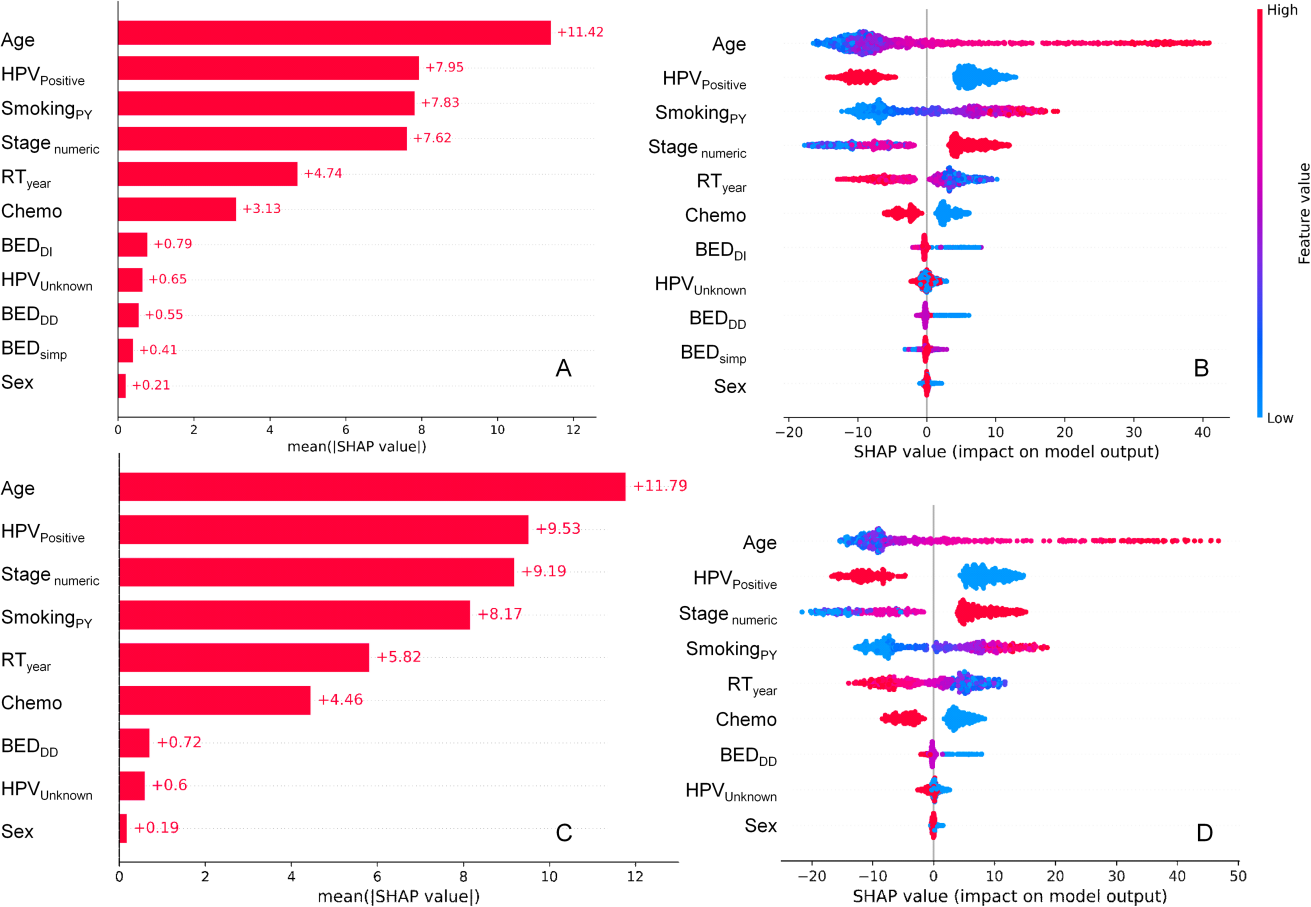
SHAP value summary plots for the RSF model for OS. **(A, B)** represent the model variant which included BED_DD_, BED_DI_ and BED_simp_, whereas **(C, D)** represent the model variant which included only BED_DD_ (not BED_DI_ or BED_simp_). **(A, C)** show mean absolute SHAP values for different features, informing abut which features contributed more or less to RSF model predictions. **(B, D)** show a detailed view where every point is a patient from the testing data set. The SHAP value scale indicates the effect on model predictions: a positive SHAP value implies an increased risk of death, while a negative value implies a reduced risk. The color of the points indicates the value of the feature for that observation, with warm colors representing higher values and cool colors representing lower values.

SHAP value summary plots for the RSF model on testing data, which show how different features contributed to the model’s predictions, are displayed in **Figures 2A, B**. Panels A and B display results for the model variant which included BED_DD_, BED_DI_ and BED_simp_, whereas panels C and D represent the model variant which included only BED_DD_ (not BED_DI_ or BED_simp_). In both models, the most important predictors of mortality in this patient population were age, HPV, stage, smoking and calendar year. Chemotherapy was the next most important predictor of mortality. Radiotherapy (BED variants) contributed less than chemotherapy, but still had non-negligible effects. Sex contributed very little to model predictions. These findings suggest that the SHAP contributions of BED_DD_ and BED_DI_ are likely to be largely redundant to each other, and it is not necessary to include both of them in the model. BED_simp_ contributed less than either BED_DD_ or BED_DI_.

**Figures 2A, C** show the mean absolute SHAP values for each feature, whereas **Figures 2B, D** provide more detail by displaying SHAP values for each individual patient in the testing data set and color coding them by feature value. For example, higher values of age (red points for the Age feature in **Figures 2C, D**) are associated with high positive SHAP values for Age, indicating that mortality risk is increased at old ages. Conversely, high red values of the HPV_Positive_ feature (which indicate HPV+ patients) were associated with negative SHAP values, indicating that HPV+ status decreased mortality risk. High values of BED_DI_ or BED_DD_ were associated with reduced mortality risk, whereas this was not obvious for BED_simp_.

Pearson correlation coefficients between all features and SHAP values in the testing data set are shown in **Supplementary Figure 1**. BED_DD_ and BED_DI_ are strongly correlated with each other and with their SHAP values, whereas the strength of correlation between them and BED_simp_ is somewhat weaker. Several other features are also strongly correlated with their own SHAP values, *e.g*. Smoking_PY_, Stage_numeric_, HPV_Positive_, Chemo, RT_year_, Age.

**Figure 3:**
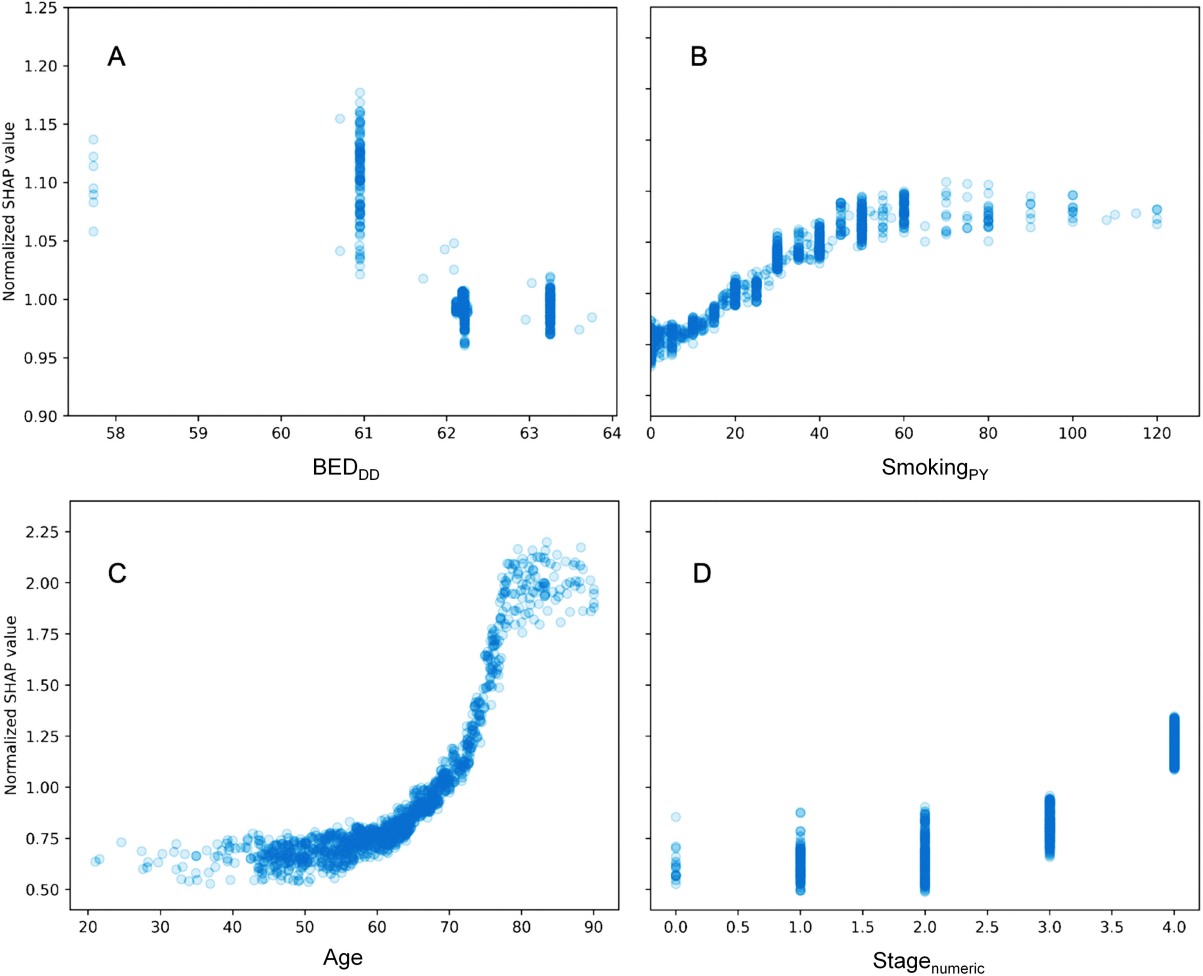
Detailed look at the relationship between some features of interest and their normalized SHAP values in the RSF model. **(A)** = BED_DD_, **(B)** = Smoking_PY_, **(C)** = Age, and **(D)** = Stage_numeric_. The y axis displays normalized SHAP values on a “relative risk” scale, where 1 represents no change from the population average, 1.1 represents a 10% increase in predicted mortality risk, and 0.9 represents a 10% decrease in predicted mortality risk.

A more detailed visualization of how the SHAP values of some features of interest are related to the feature values is provided in **Figure 3**. The y axis in each panel displays normalized SHAP values on a “relative risk” scale, where 1 represents no change from the population average, 1.1 represents a 10% increase in predicted mortality risk, and 0.9 represents a 10% decrease in predicted mortality risk. The SHAP results for BED_DD_ are shown in **Figure 3**, and those for BED_DI_ and BED_simp_ from the same model are shown in **Supplementary Figure 2**.

For BED_DD_ and BED_DI_ the patterns are very similar and nonlinear, suggesting that larger values are associated with reduced mortality. For BED_simp_, however, there is almost no change, suggesting that this BED variant which does not include repopulation is not very useful for predicting mortality. Increasing age, stage and smoking are associated with increased mortality, as expected. Notably, the y axis scales in different panels are different, since age, stage and smoking contributed more to predicting OS in the patient population, compared with the BED variants.

The competing risks RSF analysis for cause-specific mortality, using the optimized number of 300 trees, achieved reasonable concordance scores on both the training (70%) and testing (30%) portions of the analyzed data subset. Specifically, on training data its c-index values were 0.888, 0.936 and 0.863 for deaths from the index cancer, other cancers or other non-cancer causes, respectively. On testing data, its corresponding c-index values were 0.771, 0.810 and 772, respectively. The decrease in performances from training to testing was not dramatic, suggesting a relatively stable model with not much overfitting.

Visualizations of the Cause-Specific Cumulative Hazard Function (CSCHF) and Cumulative Incidence Function (CIF) for this competing risks model revealed different temporal patterns for the different causes of death (**Supplementary Figure 3**). Most deaths from index cancer (*i.e.* HNSCC recurrences) occurred within the first 5 years after treatment, whereas death hazards from other cancers and non-cancer diseases continued to increase almost linearly over the entire period of observation.

Examination of Pearson correlation coefficients between variables in this data set (**Supplementary Figure 4**), particularly between BED variants and CSCHF values for different causes of death 10 years after treatment (**Supplementary Table 1**), also revealed several interesting findings. For example, BED_simp_ variants with different α/β ratios had counterintuitive positive (rather than negative) correlations with death from index cancer. BED_DI_ variants had negative correlations, as expected, but their values were relatively small, around -0.10. BED_DD_ variants had stronger negative correlations with death from index cancer, but only for α/β ratios of 7 and 10 Gy, whereas for 13 Gy the correlation switched sign and lost its statistical significance (**Supplementary Table 1**). Overall, these results suggest that BED_DD_ and BED_DI_ have some predictive value for deaths from HNSCC recurrences and perhaps for other causes as well, but (especially for BED_DD_) there is some sensitivity to α/β ratios.

Some other variables also had interpretable behavior which conformed to expectations. For example, age was positively correlated with deaths from all causes, especially with non-cancer causes, whereas chemotherapy had expectedly negative correlations with all causes of death (**Supplementary Figure 4**). There was some variability between correlations among HNSCC diagnosis sites and cause-specific mortality, *e.g.* positive correlations for hypopharynx and negative ones for oropharynx (**Supplementary Figure 4**). These findings indicate that hypopharynx tumors were associated with higher mortality, whereas oropharynx tumors were associated with lower mortality.

### 3.2 RSF analysis of HPV negative patients

The evaluation of the RSF model for the subset of patients with HPV- status was performed as a separate analysis. Predictive performance of the RSF algorithm with tuned hyperparameters (min_samples_leaf = 10, min_samples_split = 2, n_estimators = 65) on this data subset was assessed on training data using 10-fold cross validation (CV), and separately on testing data. On training data, it generated a mean concordance score (c-index) of 0.755 on training folds and 0.634 on testing folds. On separate testing data, this model had a c-index of 0.651. This performance was somewhat worse than on the full data set likely because of reduced sample size: there were only 578 HPV- patients, *vs*. 3,346 patients in the entire data set.

**Figure 4:**
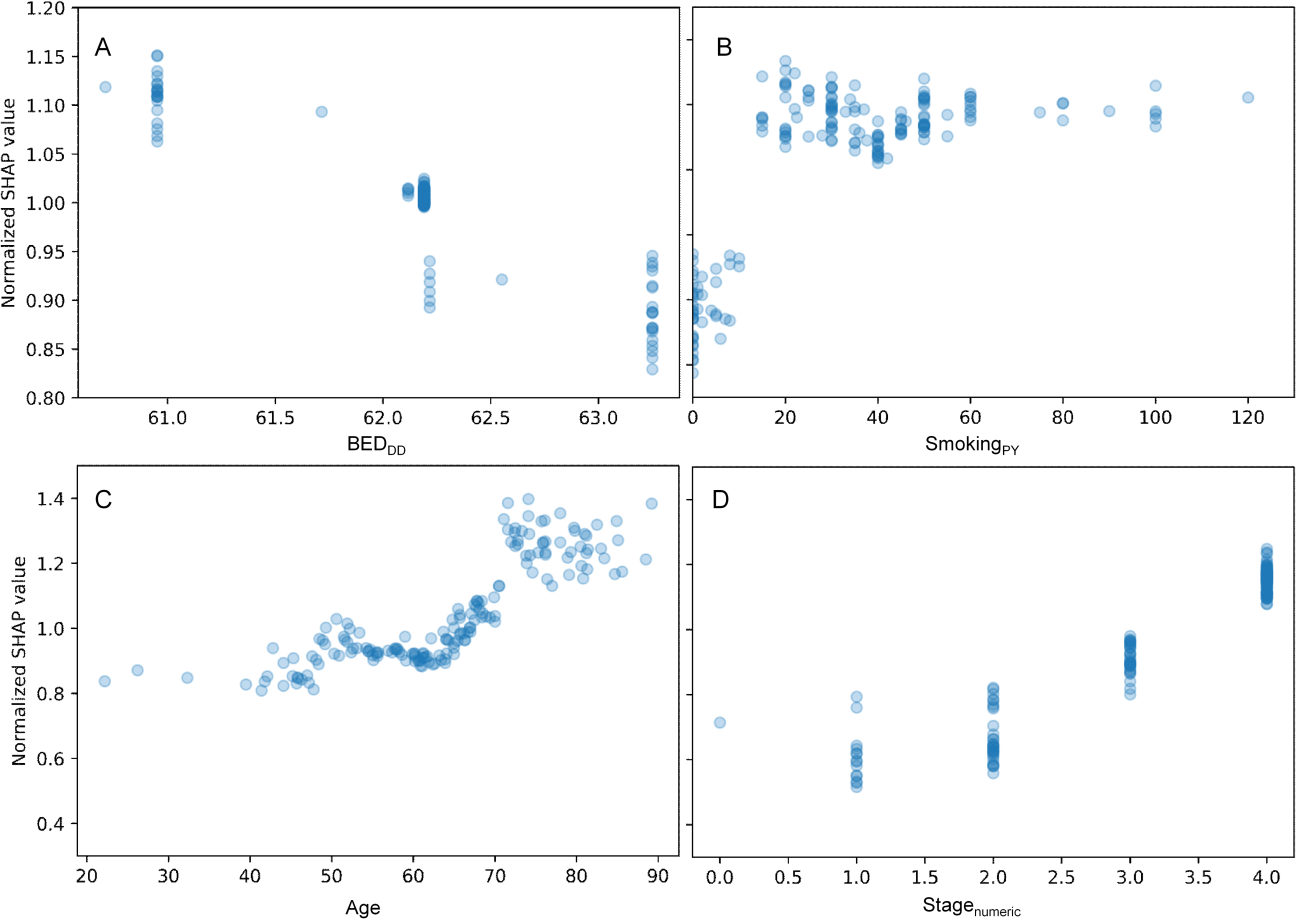
Detailed look at the relationship between some features of interest and their normalized SHAP values for the HPV- patients only. **(A)** = BED_DD_, **(B)** = Smoking_PY_, **(C)** = Age, and **(D)** = Stage_numeric_. The y axis displays normalized SHAP values on a “relative risk” scale, where 1 represents no change from the population average, 1.1 represents a 10% increase in predicted mortality risk, and 0.9 represents a 10% decrease in predicted mortality risk.

A more detailed visualization of how the SHAP values of some features of interest are related to the feature values in the HPV- patients only is provided in **Figure 4**, **Supplementary Figure 5**. The patterns were generally similar to those found for all patients (**Figure 3**, **Supplementary Figure 2**): there was a clear relationship between SHAP values and BED_DD_ and BED_DI_, but not for BED_simp_, and other variables like age, smoking and sex had major contributions. SHAP value summary plots for the RSF model for HPV- patients are shown in **Supplementary Figure 6**, and a heatmap illustrating the correlation of SHAP values among various clinical factors in the prediction model for HPV- patients is provided in **Supplementary Figure 7**. In both cases, the patterns were also not very different from those observed for all patients.

### 3.3 Causal inference CSF analysis

Following the exploratory analysis with RSF, we proceeded to the second step of our pipeline using Causal Survival Forests (CSF). This phase was aimed at conducting a targeted causal analysis, building on the patterns and predictors identified by RSF to delve deeper into their causal relationships with overall survival. Based on the RSF modeling and SHAP value analysis (**Figure 3**, **Supplementary Figure 2**), we proceeded to perform targeted causal survival forest (CSF) analyses to look at the causal effects of BED_DD_, BED_DI_ and BED_simp_ (one at a time) on OS in this patient population. To perform these analyses, BED_DD_ was converted into a binary variable by using a manual cut-point of 61.8 Gy, and the same approach was used to binarize BED_DI_ (with a cut-point of 57.6 Gy) and BED_simp_ (70 Gy). The cut-points were selected based on where a clear change in mortality prediction could be seen in the RSF SHAP values (**Figure 3**, **Supplementary Figure 2**) for BED_DD_ and BED_DI_. For BED_simp_ there was no clear change in the SHAP values as function of feature values, so the 70 Gy cut-point was selected using a similar percentile to the one used to binarize BED_DD_ and BED_DI_.

**Figure 5:**
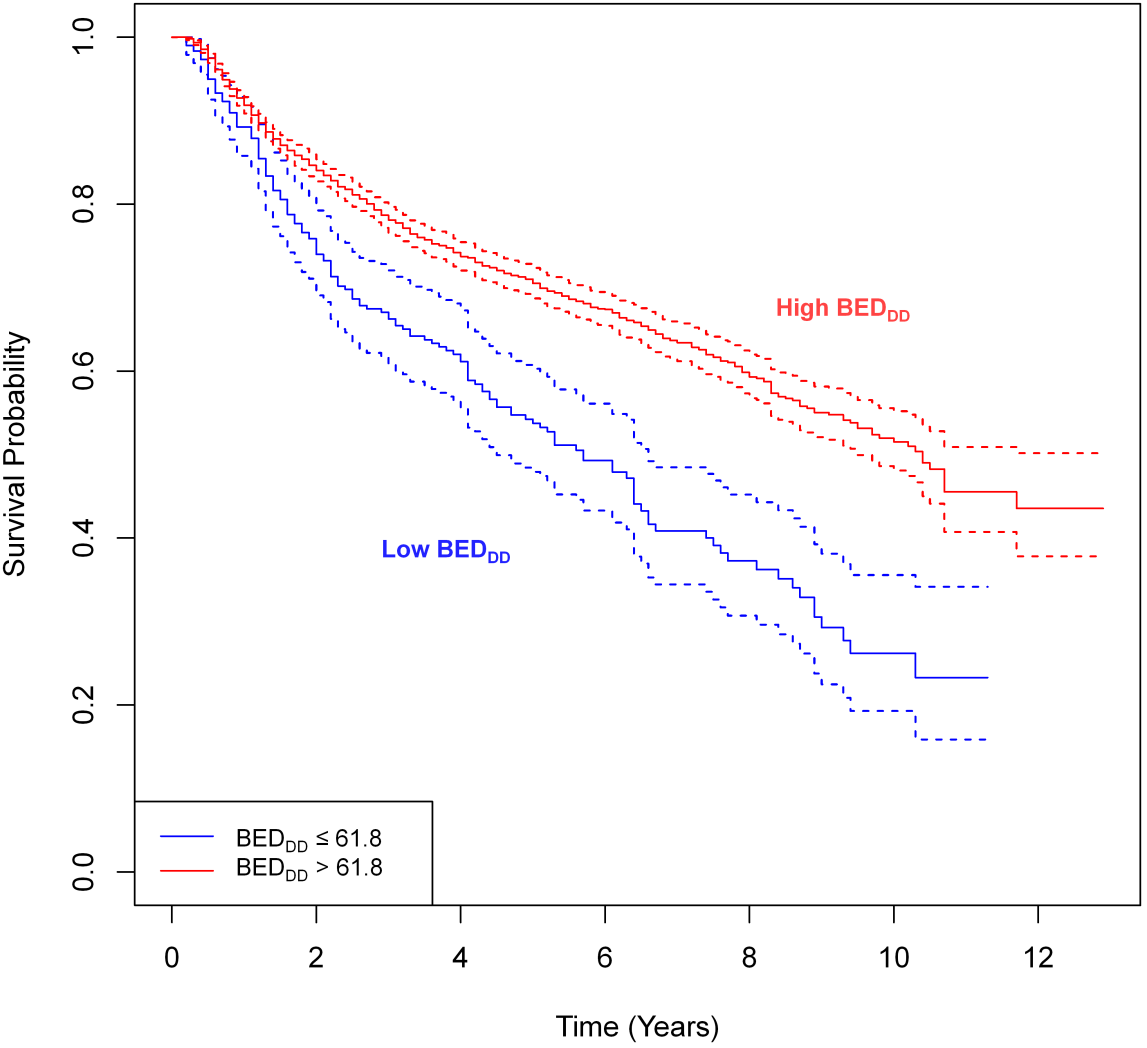
Simple univariate comparisons of Kaplan-Meier survival curves for patient groups based on splitting BED_DD_. The splitting cut-points were guided by the SHAP value analysis in the previous figure. The logrank test revealed statistically significant differences between groups: p-value = 3×10^-12^.

**Figure 5** shows simple univariate comparisons of Kaplan-Meier survival curves for patient groups split by the binarized BED_DD_. **Supplementary Figure 8** contains similar information for BED_DI_ and BED_simp_. For the BED_DD_ and BED_DI_ variants, the curves were significantly different from each other: logrank test p-value = 3×10^-12^ for BED_DD_ and p-value = 1×10^-10^ for BED_DI_. In contrast, there was no significant difference between groups split by BED_simp_ (**Supplementary Figure 8**, p-value = 0.9). If the analysis was restricted to HPV- patients only, there was still a significant difference (p-value 0.01) for BED_DD_ *vs*. low BED_DD_ despite the reduced sample size of the HPV- patient subset.

**Figure 6:**
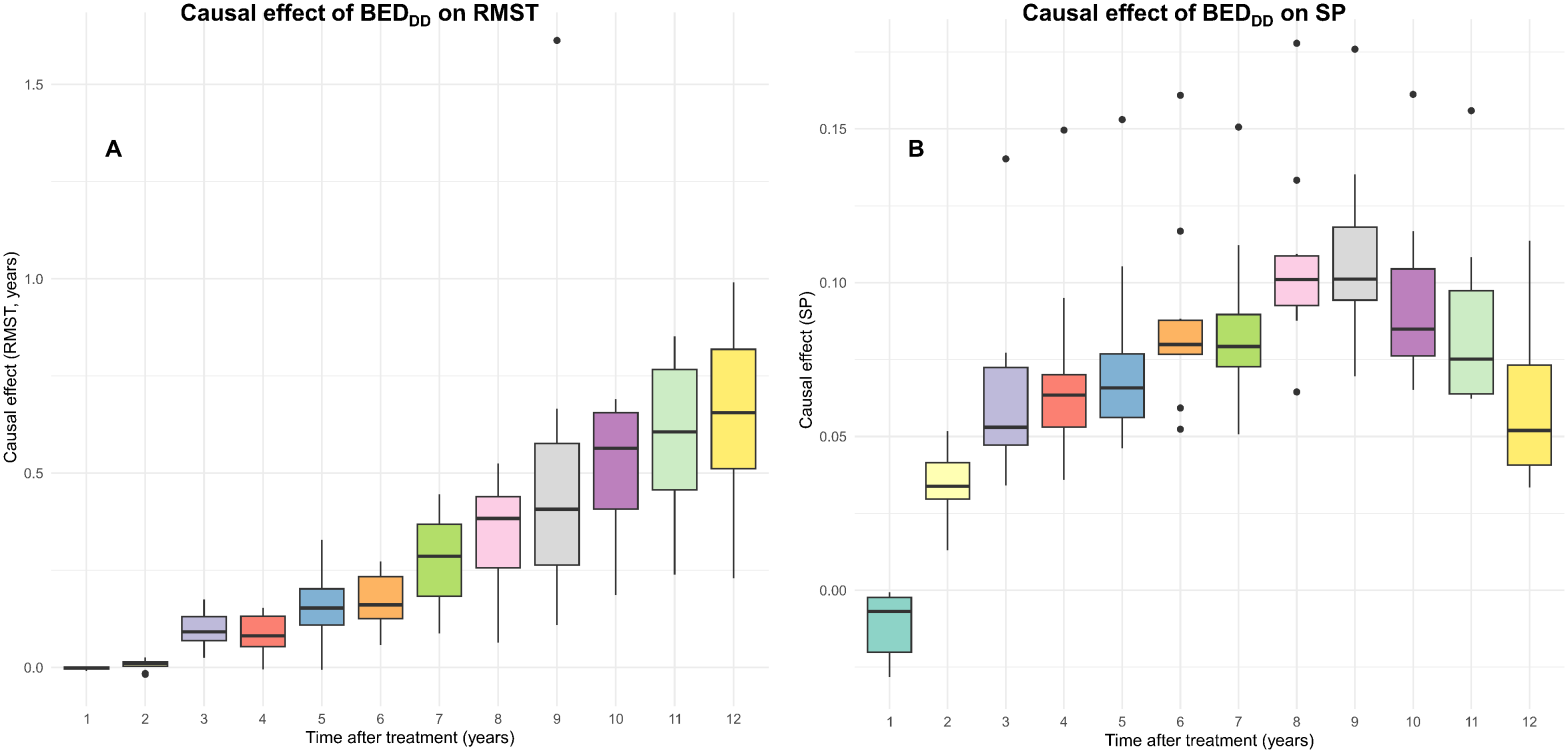
Causal effect estimates of BED_DD_ from the CSF analyses. The boxplots show the distributions of restricted mean survival time (RMST) **(A)** or survival probability (SP) **(B)** causal effects over 10-fold cross validation folds on the training data.

CSF-based causal effect (conditional average treatment effect, CATE) estimates for the effects of binarized BED_DD_, BED_DI_ and BED_simp_ on patient OS are shown in **Figure 6**, **Supplementary Figure 9**, respectively. The boxplots show the distribution of causal effect estimates for 10-fold cross validations on training data. Both the restricted mean survival time (RMST) and survival probability (SP) metrics are displayed for each BED variant at different times after treatment. These results show relatively similar patterns for BED_DD_ and BED_DI_. SP for these BED variants clearly rises above 0 starting 2 years after treatment, and reaches values of roughly 5–15%. RMST also increases steadily with time after treatment, reaching roughly 0.5–1.0 years. In other words, CSF analysis suggests that high BED_DI_ or BED_DD_ provided a clear survival advantage. However, high BED_simp_ had a different pattern: SP fluctuated around zero, and RMST dropped into the negative range at ≥5 years after treatment.

For comparison, CSF-based causal effect estimates for BED_DD_ on the entire training data set (no cross-validation) and on the entire testing data set are provided in **Table 2**. The estimates on the testing set tended to be slightly (but not dramatically) lower than on the training set. Taken together, these findings suggest that there is relatively good consistency in these effect estimates across different portions of the data, meaning that the CSF algorithm is able to generalize adequately. Several sensitivity analyses and refutation tests, described in the **Supplementary Methods**, supported this general conclusion: CSF outputs were robust to random perturbations of the data (*e.g.* injection of synthetic noise variables into the data set), did not produce spurious findings when the causal variable and survival times were randomized, but were sensitive enough to detect non-random perturbations (e.g. “fake effects” introduced into the data).

**Table 2:** Causal effect (CATE) estimates from the CSF model for different BED_DD_ for different times after treatment, on training and testing data, using the survival probability (SP) and restricted mean survival time (RMST) metrics.

Time (years)	SP (%) on training data		SP (%) on testing data	RMST (years) on training data		RMST (years) on testing data
	estimate	SE	estimate	estimate	SE	estimate
1	-1.1	1.3	-2.2	0.00	0.01	-0.01
2	3.4	1.8	2.0	0.00	0.02	-0.01
3	5.1	2.0	3.4	0.19	0.10	0.14
4	6.1	2.1	4.6	0.08	0.05	0.04
5	6.8	2.1	5.6	0.12	0.07	0.07
6	7.8	2.2	6.9	0.12	0.09	0.08
7	7.8	2.1	7.2	0.21	0.10	0.16
8	10.4	2.9	9.6	0.32	0.12	0.26
9	9.9	2.1	9.4	0.37	0.14	0.31
10	8.7	1.9	8.4	0.57	0.16	0.50
11	7.9	1.8	7.7	0.61	0.17	0.56
12	5.5	1.9	5.5	0.62	0.18	0.58

SE represents standard errors.

These causal modeling results are generally consistent with the predictive RSF modeling described above. High BED_DD_ or BED_DI_ were advantageous for OS, where BED_DI_ had a slightly stronger effect than BED_DD_. In contrast, high BED_simp_ did not provide any advantage or even a disadvantage. The likely reason is that both BED_DI_ and BED_DD_ include the important process of tumor repopulation, which is ignored in the simplistic BED_simp_.

To quantify the effects of covariates (other features) on the CSF causal effect estimates, we calculated the best linear projection (BLP) of the conditional average treatment effect. This is done by the *grf R* package by regressing doubly robust scores derived from the CSF against the covariates. The resulting uncertainty estimates are cluster- and heteroskedasticity-robust. The BLP results for the RSMT and SP metrics for BED_DD_ and BED_DI_ are shown in **Supplementary Table 2**. For the RMST metric for both BED_DD_ and BED_DI_, the only covariate which had a statistically significant effect on the RMST was stage (p-value = 7.44×10^-6^ for BED_DD_ and p-value = 0.024 for BED_DI_), and the sign of the effect was negative. Thus, higher stage of the tumor was associated with reduced RMST, meaning that the RMST advantage from high BED_DD_ or high BED_DI_ was reduced for more advanced tumors, compared with less advanced ones. For the SP metric, none of the covariate effects reached statistical significance (p-values >0.05) for both BED_DD_ and BED_DI_.

Importantly, large p-values in this analysis do not indicate that the variables have no effect on patient morality – instead, they indicate that the variables do not significantly modulate the causal effect of radiotherapy (BED) on mortality, *i.e.* that the causal effect of radiotherapy does not significantly interact with these other variables. For example, chemotherapy was clearly shown to be associated with reduced mortality – both overall (**Figure 2**) and cause-specific (**Supplementary Figure 4**) – but chemotherapy did not significantly affect the causal effect of radiotherapy (**Supplementary Table 2**), suggesting that these treatments did not detectably interact in this data set.

**Figure 7:**
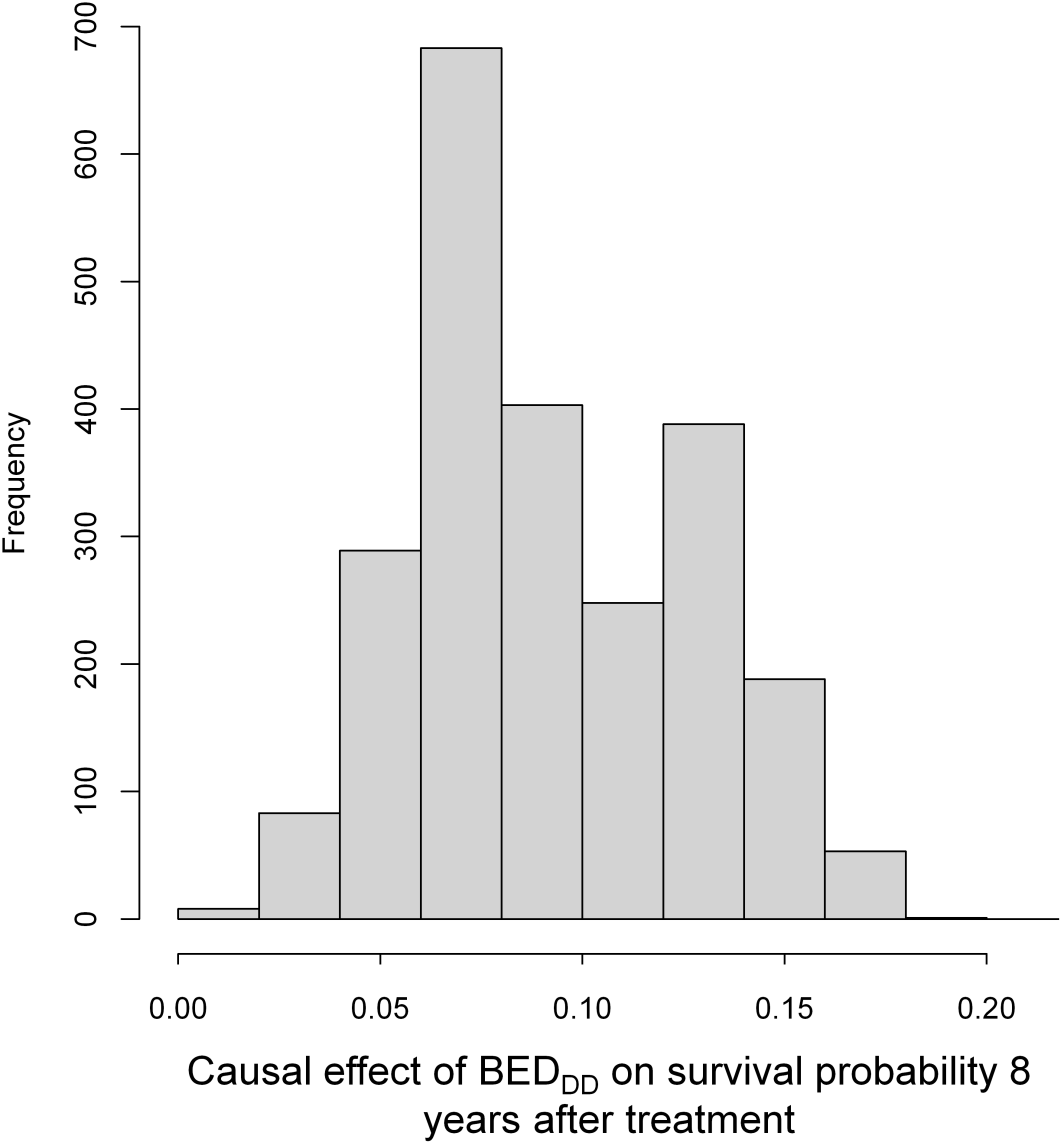
A histogram of causal effect estimates from the CSF model for individual patients (from the training data) for BED_DD_ on patient survival probability (SP). SP here was estimated at 8 years after treatment because it reached maximum values at this time point.

Importantly, in addition to generating group-averaged causal effect estimates, CSF provides effect estimates and uncertainties for each individual patient. This information can be valuable for clinicians by identifying which specific patients or patient subgroups would benefit most (or least) from the treatment, enabling improved precision medicine. A histogram of causal effect estimates from the CSF model for the effect of BED_DD_ on patient SP is shown in **Figure 7**. The figure shows that all patients had positive estimates for survival probability 8 years after treatment, *i.e.* there is an expected increase in survival for patients with high BED_DD_, vs. low BED_DD_. There is considerable variability between patients, with some expected to benefit much more than others.

## 4 Discussion

Our previous analyses using mechanistic models applied to older radiotherapy-only clinical trials data indicated that both hypofractionated schedules with doses exceeding 2 Gy/fraction and twice-daily treatments with less than 2 Gy/fraction, leading to reduced overall treatment times, significantly enhance tumor control probability (TCP) while decreasing the risk of late normal tissue complications (LNTCP) when compared to a standard 35x2 Gy protocol (14, 15). This broad conclusion held true for both the dose-independent (DI) and dose-dependent (DD) repopulation models, despite some numerical variations in predictions between the models. The effectiveness of hypofractionation and twice-daily hyperfractionation is rooted in their shared radiobiological impact, intensifying tumor cell killing per day, a pattern consistent irrespective of the repopulation model details. Thus, hypofractionation and its accelerated hyperfractionation variant prove to be efficient strategies for overcoming tumor repopulation, particularly beneficial for fast-growing tumors like HNSCC. Recent clinical evidence indeed supports the superiority of hyperfractionation over standard fractionation^13,38^ and ongoing clinical trials are now exploring hypofractionation, benefiting from advances in radiation delivery techniques that mitigate acute toxicity concerns (7).

This paper extends our initial studies (14, 15) by leveraging contemporary data from the RADCURE project (2005–2017), encompassing 3,346 HNSCC patients. Through advanced machine learning methods, we aimed to assess the impact of radiotherapy fractionation on overall patient survival more comprehensively. This research combined three radiobiological modeling approaches: (1) Implementing the mechanistic concept of biologically effective dose (BED) for the standard dose-independent (DI) tumor repopulation model, the alternative dose-dependent (DD) repopulation model, and a simple model with no repopulation (BED_simp_). (2) Employing a robust predictive machine learning algorithm, random survival forests (RSF), to model patient overall survival (OS) and cause-specific mortality using the BED variants alongside clinical variables. (3) Conducting targeted causal inference analyses using the CSF algorithm to estimate the causal effect of each BED variant separately on OS. This cohesive approach allowed us to first identify key predictors of survival using RSF in a broad analysis and then employ CSF to rigorously investigate the causal impact of these variables. By separating the exploratory and causal phases, we were able to provide a more nuanced understanding of the data, highlighting not only the importance of certain predictors but also their causal relationships with survival outcomes. This methodology underscores the potential of combining predictive and causal machine learning techniques to enhance the interpretation and application of clinical data.

Moreover, by providing individual-level causal effect estimates, the research aligns with contemporary movements towards personalized medicine, offering a valuable framework for customizing radiotherapy strategies to patient-specific characteristics. However, the study’s strengths are tempered by certain limitations. Interpretability of sophisticated machine learning techniques such as RSF and CSF is generally more difficult, than for simpler models such as Cox regression, which are more common in the medical literature. Additionally, the study’s observational nature suggests the potential for unmeasured confounding factors that could influence the conclusions drawn. Despite employing advanced causal inference techniques designed to address such concerns, the possibility of residual confounding cannot be entirely dismissed, highlighting an area for cautious interpretation and further investigation. Importantly, this study is also limited because it did not directly assess radiotherapy-related toxicity, which can be affected by altered fractionation schemes. Unfortunately, modeling toxicity here was not possible since toxicity data were not included in the RADCURE data set. A separate validation data set was not used for the study, although a random training/testing split of the large RADCURE data set was performed. As commonly recommended in machine learning analyses, all model optimization and fitting was performed on the training portion, while the testing portion was not shown to any model until the evaluation stage. Finally, the RADCURE data set was constructed using the outdated 7-th edition American Joint Committee on cancer (AJCC) tumor classification, which does not separate some important predictors of tumor recurrence, such as number of nodes, nodal location (high *vs*. low neck nodes), which also results in a limitation on our study.

Aligning with our earlier hypotheses, the current study’s findings emphasize the importance of optimizing fraction size to mitigate tumor repopulation. This study, employing advanced machine learning on a contemporary clinical dataset, reinforces the potential benefits of increased intensity of tumor killing per day in improving HNSCC treatment. Specific examples of comparing BED_DD_ and other BED variants for several treatment regimens present in the RADCURE data set, and for some hypothetical ones, are provided in **Table 3**. For example, 25×2.4 Gy, 20×2.75 Gy, and 18×3.0 Gy regimens increase BED_DD_ and BED_DI_ relative to standard fractionation (**Table 3**) and may represent potentially clinically useful hypofractionated options for treating HNSCC.

**Table 3:** Specific examples of comparing BED variants for several treatment regimens present in the RADCURE data set, and for some hypothetical ones (marked by *).

Number of fractions	Dose/fraction (Gy)	Total dose (Gy)	Treatment duration (weeks)	BED_DD_		BED_DI_		BED_simp_	
				value	Differrence with standard	value	Differrence with standard	value	Differrence with standard
38	1.8*^B^	68.4	3.8	65.68	3.49	75.13	10.96	80.71	-3.29
18	3*	54	3.6	63.86	1.67	64.84	0.67	70.20	-13.80
20	2.75	55	4	63.60	1.41	64.32	0.15	70.13	-13.88
25	2.4	60	5	63.25	1.06	64.80	0.63	74.40	-9.60
20	2.6	52	4	63.03	0.84	59.72	-4.46	65.52	-18.48
21	2.55	53.55	4.2	62.95	0.76	60.53	-3.65	67.21	-16.79
30	2.2	66	6	62.90	0.71	65.81	1.63	80.52	-3.48
22	2.4	52.8	4.4	62.72	0.53	58.06	-6.11	65.47	-18.53
60	1.1^B^	66	6	62.33	0.13	58.55	-5.63	73.26	-10.74
*35*	*2*	*70*	*7*	*62.19*	*0.00*	*64.17*	*0.00*	*84.00*	*0.00*
40	1.6	64	8	60.95	-1.24	49.30	-14.87	74.24	-9.76
25	2	50	5	57.73	-4.46	50.40	-13.77	60.00	-24.00
40	1.27	50.8	4	55.46	-6.73	51.45	-12.73	57.25	-26.75

B indicates BID (twice daily) fractionation, whereas all other regimens use QD (once daily) fractionation. The italicized row indicates the standard regimen.

Acute toxicity from the more intense treatments can be manageable under modern dose delivery techniques. For example, in a multi institutional phase II study of concomitant stereotactic reirradiation and cetuximab for recurrent head and neck cancer, reirradiation with 36 Gy in six fractions of 6 Gy (*i.e.* 6 Gy/fraction) to the 85 isodose line covering 95 of the PTV with 5 injections of concomitant cetuximab still resulted in acceptable acute toxicity levels^39^. We believe that our study contributes to the growing body of knowledge on the role of RT fractionation in head and neck cancer treatment, offering valuable insights for clinicians and researchers alike.

## Data Availability

Publicly available datasets were analyzed in this study. This data can be found here: https://www.cancerimagingarchive.net/collection/radcure/.
