# Supplementary material for: Understanding the impact of radiotherapy fractionation on overall survival in a large head and neck squamous cell carcinoma dataset: a comprehensive approach combining mechanistic and machine learning models
Source: Front Oncol. 2024 Aug 13;14:1422211. doi: 10.3389/fonc.2024.1422211 (PMC11347346; doi:10.3389/fonc.2024.1422211)
Supplement: Supplementary file 1 [file DataSheet1.docx]

Supplementary Material

Understanding the Impact of Radiotherapy Fractionation on Overall Survival in a Large Head and Neck Squamous Cell Carcinoma Dataset: A Comprehensive Approach Combining Mechanistic and Machine Learning Models

Igor Shuryak*, Eric Wang, David J. Brenner

*** Correspondence:** Igor Shuryak: is144@cumc.columbia.edu

# Supplementary Methods

**Data Collection**

The dataset for analysis was obtained from the RADCURE project, consisting of 3,346 head and neck cancer patients treated with definitive radiotherapy (RT) at the University Health Network in Toronto, Canada^1,2^. The dataset furnishes demographic, clinical, and treatment information, featuring a median patient age of 63, predominantly comprising males. Oropharyngeal cancer accounts for 50% of cases, while larynx, nasopharynx, and hypopharynx cancer constitute 25%, 12%, and 5%, respectively. The radiotherapy details (RTSTRUCTs) adhere to the TG263 nomenclature, incorporating anonymization measures. This dataset holds potential utility in quantitative image analysis research, machine learning applications, and the development of prognostic models within the realm of head and neck cancer. It encompasses CT images, demographic details, clinical and treatment information, and survival outcomes. Notably, this analysis exclusively leveraged the tabular data and did not involve the utilization of images.

The RADCURE dataset is very useful for investigating the effects of different radiotherapy fractionation schemes on HNSCC patient survival because it includes a variety of total doses and doses per fraction. The summary statistics for total dose (D) were: Range: 50-74 Gy, Median: 70.00, Mean: 66.66. For dose/fraction (d) they were: Range: 1.10-2.75 Gy, Median: 2.00, Mean: 2.05; and for number of fractions (m) they were: Range: 20-60, Median: 35.00, Mean: 33.02. Total duration of treatment (T) was not explicitly reported in the data set, so we calculated it assuming that treatment was only given on weekdays with gaps on weekends, that irradiations were performed once per weekday for d >1.5 Gy and twice per weekday for d ≤1.5 Gy.

**Data Preprocessing and Feature Selection**

The RADCURE data set contained numerous clinical variables, and for analysis we selected the following most relevant ones, trying to avoid redundancy: Age (patient age, years), Sex (0=female, 1=male), Smoking_PY_ (number of packs smoked in a year), Stage_numeric_ (AJCC 7th edition staging categories, converted into integers of 0-4), HPV (tumor HPV status determined by p16 IHC +/- HPV DNA by PCR), Chemo (1=received concurrent chemoradiotherapy, 0=did not receive concurrent chemoradiotherapy), RT_year_ (calendar year of the radiotherapy treatment), Status (binary indicator of vital status at last contact date), Length_FU_ (duration of follow up from diagnosis to last contact date in years). The HPV status was categorical, indicating positive, negative, or unknown (missing). To incorporate this information into our analysis, we applied one-hot encoding, representing HPV- as the default, while creating separate binary columns for HPV_Positive_ and HPV_Unknown_. The Status and Length_FU_ variables were the outcome variables, indicating overall survival (OS).

The data were imported and analyzed using the *R* and Python programming languages. Missing values in important variables were imputed using the *MissForest* algorithm in *R*, and categorical variables were one-hot encoded into several binary columns to prepare the dataset for machine learning analysis. The percentages of missing values were small: 1.49 for Smoking_PY_, 0.81 for Stage, and zero for the other variables. To perform subsequent machine learning analyses described below, we split the data set randomly, with 70% designated for training and the remaining 30% for testing.

**Calculation of Biologically Effective Dose (BED) for Different Tumor Killing and Repopulation Models**

To compare the effects of various radiotherapy regimens present in the RADCURE data set on patient OS, we employed the well-established Biologically Effective Dose (BED) concept^3^. If tumor repopulation is neglected, a simplistic BED can be calculated as follows, where m is the number of fractions, d is dose per fraction, and r is the α/β ratio (assumed to be 10 Gy for HNSCC):

$BED_{simp}=m d (1+\frac{d}{r})$ (1)

A more advanced BED version which includes accelerated tumor repopulation (AR) which is assumed to begin at a fixed onset time T_k_ is based on the work of Withers et al^4^. Since T_k_ is assumed to be independent of radiotherapy details such as dose or dose/fraction, we called this the “dose independent” (DI) model, and the consequent BED is labeled BED_DI_. Based on our previous publication^5^, the equation for BED_DI_ can be derived using the following steps.

Under the DI model, the natural logarithm of total tumor cell killing (lnS_DI_) by the radiotherapy regimen consisting of m fractions is described by the following equation, where λ = accelerated tumor repopulation rate, g = background slow repopulation rate (not accelerated), T_k_ = onset time for accelerated repopulation, T = total radiotherapy treatment time:

$lnS_{DI}=-m\left( \alpha d+\frac{\alpha d^{2}}{r} \right)+gT+\lambda max(0,T-Tk)$ (2)

To convert this to BED for the DI model (BED_DI_), one needs to use the relationship BED_DI_ = - lnS_DI_/α, which leads to the following formula:

$\mathrm{BED}_{\mathrm{DI}}=[m \alpha d\frac{d + r}{r}- g T - \lambda max\left( 0, T - T_{k} \right)]/\alpha$ (3)

As another alternative, we considered our proposed “dose dependent” (DD) tumor repopulation model, where both the onset time and rate of AR are assumed to depend on the average fraction of tumor cells killed by radiotherapy each day^5^. In other words, in this model the tumor responds to a higher “intensity” of tumor cell killing by radiotherapy by starting AR earlier and increasing its rate. AR is assumed to begin when the natural logarithm of the tumor cell surviving fraction drops below a certain value –C. The following steps can be used to derive BED for the DD model (BED_DD_).

The average number of dose fractions per day is m/T, so then the average surviving fraction of cells per day (surv_av_) is:

$surv_{av}=e^{-\frac{m\left( \alpha d+\left( \frac{\alpha d^{2}}{r} \right) \right)}{T}}$ (4)

Under the DD model, accelerated repopulation is assumed to start at time T_k_DD. This critical time is defined as the time after the start of treatment when the natural logarithm of the tumor cell surviving fraction decreases below a threshold level –C. This condition can be expressed as:

$-C=TkDD ln[surv_{av}]= -\frac{mT_{k}DD\left( \alpha d +\frac{\alpha d^{2}}{r} \right)}{T}$ (5)

The solution for T_k_DD is:

$T_{k}DD=\frac{CTr}{m\alpha d(d+r)}$ (6)

These calculations provide the onset time for accelerated repopulation under the DD model. The rate of accelerated repopulation is given by the expression λ (1-surv_av_), where 1-surv_av_ represents the average fraction of tumor cells killed per day by radiotherapy.

These components of the DD model are then combined to calculate the natural logarithm of total tumor cell killing (lnS_DD_):

$lnS_{DD}= -m\left( \alpha d+\frac{\alpha d^{2}}{r} \right)+gT+\lambda\left( 1-e^{-\frac{m\left( \alpha d+\frac{\alpha d^{2}}{r} \right)}{T}} \right)max(0,T-\frac{CTr}{m\alpha d\left( d+r \right)})$ (7)

To convert this equation to BED for the DD model (BED_DD_), one needs to use the relationship BED_DD_ = - lnS_DD_/α, which leads to the following formula:

$\mathrm{BED}_{\mathrm{DD}}=\frac{\left[ r \lambda\left( \exp\left( -m \alpha d\frac{d + r}{T r} \right)- 1 \right)\max\left( 0, -T\frac{-m \alpha d \left( d + r \right)+ C r}{m \alpha d \left( d + r \right)} \right)+ \left( \alpha d m - T g \right) r + m \alpha d^{2} \right]}{r \alpha}$ (8)

The parameters were taken from reference ^5^. They were as follows: BED_DI_: α = 0.069 Gy^-1^, λ = 0.035 days^-1^, T_k_ = 28.6 days; BED_DD_: α = 0.224 Gy^-1^, λ = 1.17 days^-1^, C = 14.5^5^. The α/β ratio r was 10 Gy for both models. The resulting BED_simp_, BED_DI_ and BED_DD_ variables were included in the RADCURE data set as predictors of OS.

**Predictive Machine Learning Analysis using Random Survival Forests (RSF)**

We utilized Random Survival Forest (RSF), a predictive machine learning method, to model OS using all the other available variables (features) in the data set. The goals of this analysis were to identify: (1) How accurately can OS be predicted using the available variables? (2) Which variables are most important contributors to these predictions? (3) How does radiotherapy, represented by the BED_DD_, BED_DI_ and BED_simp_ variables, contribute to predicting OS?

RSF is a machine learning method tailored for analyzing right-censored survival data, an extension of Leo Breiman's random forest method ^6^. Here we implemented RSF using the sksurv.ensemble RandomSurvivalForest Python package. It constructs an ensemble of tree-based learners, each built on a different bootstrap sample of the original training data. At each node, the split criterion is evaluated only for a randomly selected subset of features and thresholds, which helps de-correlate individual trees. Predictions are formed by aggregating predictions of individual trees in the ensemble. RSF can capture complex relationships between predictors and survival without requiring prior specification and has superior predictive performance. It introduces new survival splitting rules for growing survival trees. RSFs provide a robust and flexible method for analyzing survival data, handling complex data structures, and providing interpretable results, making them particularly useful in medical research and other fields where understanding time-to-event outcomes is crucial.

The RSF model was optimized using grid search for hyperparameter tuning, ensuring robust performance. In this tuning process, a parameter grid was defined, specifying different values for the number of estimators, minimum samples to split, and minimum samples per leaf. The RSF model was then instantiated, and a GridSearchCV object was created with 10-fold cross-validation to explore various combinations of hyperparameters. The grid search was performed in parallel to efficiently search the hyperparameter space. After fitting the GridSearchCV object to the training data, the best hyperparameters were identified. The best model was then obtained using these optimal hyperparameters. Finally, concordance scores were calculated for both the training and testing datasets to evaluate the performance of the tuned RSF model. The goal was to find the hyperparameter configuration that maximized the concordance scores, indicating better predictive accuracy for survival outcomes.

**SHapley Additive exPlanations (SHAP) Value Calculation and Analysis for the RSF Model**

SHAP values are a state of the art method for interpreting complex machine learning models, such as those used here. SHAP values^7^, a versatile interpretability method, play a crucial role in comprehending the influence of individual features on the predictions made by machine learning models. Their utility extends to providing local interpretability by elucidating how each feature impacts a specific prediction, and they are adaptable across various algorithms. SHAP values adhere to the principles of additivity and consistency, imparting a user-friendly understanding of feature significance. They facilitate both local and global interpretation, revealing feature interactions and aiding users in making informed decisions, assessing model reliability, and troubleshooting model issues. Visual representations of SHAP values offer clear insights into model behavior and individual predictions, thereby enhancing transparency and explainability in the domain of machine learning.

Here we calculated SHAP values for all features in the RSF model and visualized them. Normalized SHAP values were calculated for each feature by adding the base values and SHAP values and then dividing the result by the base values. This approach facilitates the interpretation of the impact of each feature on the model's predictions relative to their baseline values. In essence, the normalized SHAP values are placed on a relative risk (RR) scale, where, for example, 1.1 represents a 10% increase in mortality risk, whereas 0.9 represents a 10% decrease in mortality risk.

**Causal Machine Learning Analysis Using Causal Survival Forest (CSF)**

The CSF algorithm, implemented using the causal_survival_forest of the *grf R* package^8,9^, was employed to quantify the *causal* effects of each BED variant (BED_DD_, BED_DI_ or BED_simp_) on OS. CSF is a subtype of Causal Forests (CF)^9-12^, which are a machine learning method used to estimate treatment effects in observational data. They aim to understand how different variables (or “treatments”) causally affect an outcome. The training data for CF analysis is split into two halves: one for growing the trees and determining their structure (the “splitting” set), and the other for estimating treatment effects (the “estimation” set). This is a process known as “honesty” in the context of random forests. In this method, for each training sample the algorithm only uses the response (outcome variable) to estimate the within-leaf treatment effect or to decide where to place the split, but not both. The prediction of treatment effects is the difference in the average outcomes between the treated and the control observations of the estimating subsample in terminal leaves.

The CSF algorithm uses a binary (0 or 1) causal/treatment variable as the input. Consequently, we converted continuous BED_DD_, BED_DI_ or BED_simp_ into binary variables by using manually defined cut-points guided by the SHAP analysis results from the RSF model described above. For example, the examination of RSF-generated SHAP values for BED_DD_ suggested a nonlinear response, where a clear reduction in patient mortality was associated with BED_DD_ values >61.8 Gy. Consequently, a causal variable for CSF analysis was created where BED_DD_ values ≤61.8 Gy were mapped to 0, and BED_DD_ values >61.8 Gy were mapped to 1. The same approach was used to binarize BED_DI_ and BED_simp_, but with different cutoff values.

Consequently, three separate CSF analyses were performed, using binarized versions of either BED_DD_, BED_DI_ or BED_simp_ as the causal/treatment variable. In each case, the other two BED versions were not included in the data set. For example, if binarized BED_DD_ was the causal variable, BED_DI_ and BED_simp_ were not included. The set of covariates/potential confounders was the same in each analysis: Sex, Smoking_PY_, Stage_numeric_, HPV_Positive_, HPV_Unknown_, Chemo, RT_year_, Age.

Two estimands (metrics) were selected to describe the causal effect of each BED variant. They were RMST (Restricted Mean Survival Time) and survival probability (SP). RMST represents the average survival time up to a specific time point (e.g., a fixed follow-up time or a certain event occurrence). Notably, it offers a straightforward and easily communicable representation of the average survival duration up to a specified time point. Its significance is particularly pronounced in clinical research, as it provides a clinically comprehensible summary of time-to-event data, facilitating the evaluation of intervention effectiveness and therapeutic outcomes. Additionally, RMST exhibits robustness in the face of violations of the proportional hazards assumption, rendering it well-suited for diverse study scenarios where alternative methods may prove less effective. In comparison, SP refers to the likelihood of an event (death in this case) occurring beyond a given time point. In causal survival forests, SP provides insights into the probability of survival (or event-free survival) at specific time intervals. Both RMST and SP were calculated for various times after treatment.

**CSF Sensitivity Analysis and Refutation Tests**

Since the true causal effects are of course unknown during analyses of observational data such as those performed here, *in silico* sensitivity analyses and refutation tests are crucial in evaluating the robustness of CML models. They help verify the reliability of causal assumptions and the model's sensitivity to potential confounding. These tests are particularly vital when true causal effects are indeterminate, and the assumption of no hidden confounding is likely imperfect, which is common in real-world data scenarios.

One of the steps in CSF implementation involves making a separate model for predicting “treatment propensities” (W.hat) based on the covariates. In this case, W.hat represents the probability for a given patient to have 1 instead of 0 as the causal variable value. For example, if the causal variable is BED_DD_, W.hat is the probability to have BED_DD_ > cut-point. The default method in the *grf R* package uses a regression forest for predicting W.hat, but in this data set it yielded values that were extremely close to the boundaries of 0 or 1, which can destabilize the causal effect estimates from CSF. To address this issue, we used a regularized elastic net regression model (implemented by the *glmnet R* package) instead of regression forest to predict W.hat.

The goal of regularized regression is to prevent overfitting and improve the model's generalization performance. Regularization adds a penalty term to the traditional regression objective function, which includes the sum of squared errors. This penalty discourages the regression coefficients from reaching excessively large values, thus preventing the model from becoming too complex and fitting noise in the training data. In L1 regularization, the penalty term is proportional to the absolute values of the coefficients, encouraging sparsity by setting some coefficients exactly to zero. This leads to feature selection, as only a subset of the features contributes to the model. In L2 regularization, the penalty term is proportional to the square of the coefficients, preventing any single coefficient from dominating the objective function. It controls the overall size of the coefficients. Elastic Net combines both L1 and L2 regularization, providing a balance between feature selection and coefficient shrinkage. In elastic net, alpha represents the elastic net mixing parameter, determining the balance between L1 (lasso) and L2 (ridge) regularization. Lambda is the regularization parameter controlling the strength of regularization.

To implement elastic net on our data, a sequence of alpha values (controlling the mix of L1 and L2 regularization) over a broad range was created. The code iterated through each alpha value, performing cross-validation using the cv.glmnet function with a binomial family, class type measure, and 10-fold cross-validation. The optimal lambda values for each alpha were extracted, and the alpha with the lowest cross-validated error was identified. The final model was trained using the selected alpha and its corresponding optimal lambda, incorporating elastic net regularization, a combination of L1 and L2 regularization. W.hat values were predicted for the training and testing data sets. To further ensure the stability and reliability of our causal inference, we imposed an additional constraint on the W.hat values, truncating them to fall within the range of W.hat to fall between 0.1 and 0.9.

The resulting optimal elastic net model with range adjustment was used to predict W.hat on training and testing data, and these W.hat values were passed to the CSF algorithm. To test the stability of the RMST and SP estimates from CSF, we fitted it with 10-fold cross validation on training data, and also made a separate evaluation on testing data.

As an additional level of testing, we performed CATE (Conditional Average Treatment Effect) refutation tests. They are used in the causal inference to assess the validity of assumptions and models in estimating treatment effects. The purpose of CATE refutation tests is to evaluate the robustness of causal inference methods and the underlying assumptions by examining whether observed data conform to the expected patterns implied by the assumed causal model. The key idea behind CATE refutation tests is to simulate hypothetical interventions or treatments and compare the predicted outcomes under these interventions to the observed outcomes. If the assumed causal model is correct, the observed data should align with the expected patterns.

Specifically, we used the following refutation tests. *Dummy outcome* test, where both the causal variable and the death times in the data are randomly shuffled (or replaced with vectors of random numbers) multiple times, and the expected CSF output would be a distribution of causal effects around zero. *Random perturbation (increase) of censoring probabilities*, where some of the observed death events are randomly converted into censored observations, and the expected output is relatively unperturbed causal effect estimates, but with larger standard errors. *Fake effects* tests, where the outcome (death times), or both the outcome and causal variable, are modified in a known way, *e.g.* by linear functions with certain “fake” effect sizes. Here the expected output is to shift the causal effect estimates in the direction of the “fake” effect, based on its magnitude. *Noise variable tests*, where several (*e.g.* 5) synthetic noise variables (random numbers drawn from the standard Normal distribution) are added to the data set, and the expected outcome is no appreciable effect on the causal effect estimates.

# Supplementary Figures and Tables

**Supplementary Figure 1.** Pearson correlations matrix of all features and their SHAP values on the testing data set.

**Supplementary Figure 2.** Detailed look at the relationship between some features of interest and their normalized SHAP values from the RSF model. Left panel = BED_DI_, and right panel = BED_simp_. The y axis displays normalized SHAP values on a “relative risk” scale, where 1 represents no change from the population average, 1.1 represents a 10% increase in predicted mortality risk, and 0.9 represents a 10% decrease in predicted mortality risk.

**Supplementary Figure 3**. Cause-Specific Cumulative Hazard Function (CSCHF) and Cumulative Incidence Function (CIF) plots for deaths from the index cancer (black), other cancers (red) or non-cancer causes (green). These functions were generated by the RSF model with competing risks on the training data portion of the subset analysis described in the main text.

**Supplementary Figure 4**. Pearson correlation coefficient matrix for all variables in the training data portion of the subset analysis described in the main text. Variables are listed in alphabetical order. Death_from_index_cancer, Death_from_other_cancer, and Death_from_other_cause represent the CSCHF values for different causes of death 10 years after treatment predicted by the competing risks RSF model. BED variants were constructed using the DD, DI and simp formulae described in the main text Methods section, with the numbers 7, 10 and 13 representing α/β ratio values of 7, 10 and 13 Gy, respectively.

| **Variable** | **Death from index cancer** | | **Death from other cancer** | | **Death from other (non-cancer) cause** | |
| --- | --- | --- | --- | --- | --- | --- |
|  | **r** | **p** | **r** | **p** | **r** | **p** |
| **BED _DD 10_** | -0.21 | 0.000 | 0.06 | 1.000 | 0.08 | 0.336 |
| **BED _DD 13_** | 0.08 | 0.297 | -0.11 | 0.000 | 0.05 | 1.000 |
| **BED _DD 7_** | -0.25 | 0.000 | 0.10 | 0.003 | 0.06 | 1.000 |
| **BED _DI 10_** | -0.10 | 0.002 | -0.16 | 0.000 | -0.15 | 0.000 |
| **BED _DI 13_** | -0.10 | 0.003 | -0.16 | 0.000 | -0.15 | 0.000 |
| **BED _DI 7_** | -0.11 | 0.001 | -0.16 | 0.000 | -0.14 | 0.000 |
| **BED _simp 10_** | 0.16 | 0.000 | -0.31 | 0.000 | -0.22 | 0.000 |
| **BED _simp 13_** | 0.17 | 0.000 | -0.31 | 0.000 | -0.22 | 0.000 |
| **BED _simp 7_** | 0.15 | 0.000 | -0.31 | 0.000 | -0.22 | 0.000 |

**Supplementary Table 1.** Pearson correlation coefficients (r) and Bonferroni-adjusted p-values (p) between BED variants and CSCHF values for different causes of death 10 years after treatment predicted by the competing risks RSF model. These BED variants were constructed using the DD, DI and simp formulae described in the main text Methods section, with the numbers 7, 10 and 13 representing α/β ratio values of 7, 10 and 13 Gy, respectively.

**Supplementary Figure 5.** Detailed look at the relationship between some features of interest and their normalized SHAP values from the RSF model for HPV- patients only. Left panel = BED_DI_, and right panel = BED_simp_. The y axis displays normalized SHAP values on a “relative risk” scale, where 1 represents no change from the population average, 1.1 represents a 10% increase in predicted mortality risk, and 0.9 represents a 10% decrease in predicted mortality risk.

**Supplementary Figure 6.** SHAP value summary plots for the RSF model for HPV- patients. The left panel shows mean absolute SHAP values for different features, informing abut which features contributed more or less to RSF model predictions. The right panel shows a detailed view where every point is a patient from the testing data set. The SHAP value scale indicates the effect on model predictions: a positive SHAP value implies an increased risk of death, while a negative value implies a reduced risk. The color of the points indicates the value of the feature for that observation, with warm colors representing higher values and cool colors representing lower values.


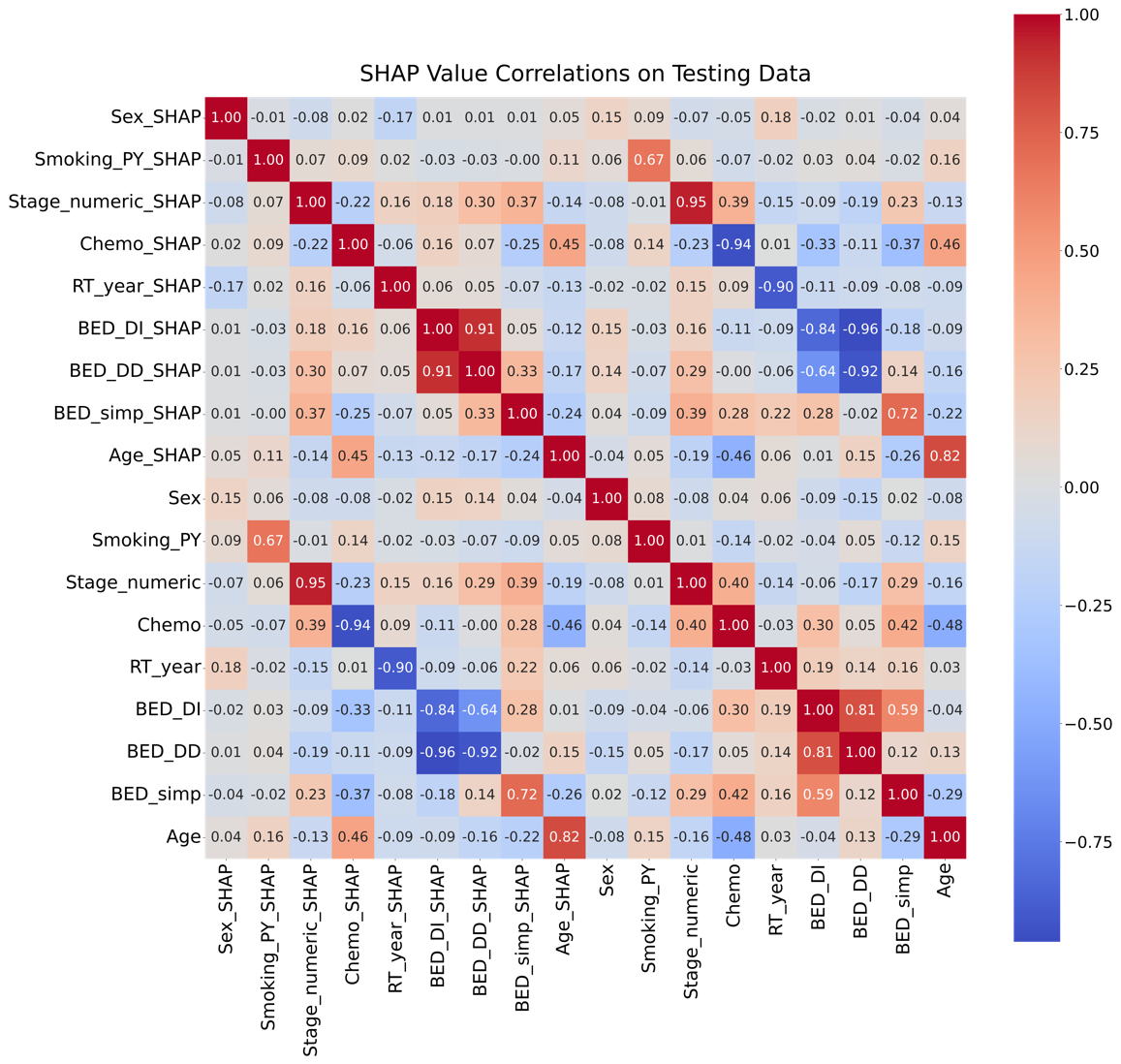


**Supplementary Figure 7.** Heatmap illustrating the correlation of SHAP values among various clinical factors in the RSF model for HPV- patients. Red and blue colors indicate positive and negative correlations, respectively.

**Supplementary Figure 8.** Simple univariate comparisons of Kaplan-Meier survival curves for patient groups based on splitting BED_DI_ (panel A) or BED_simp_ (panel B). The splitting cut-points were guided by the SHAP value analysis in the previous figure. Logrank tests revealed statistically significant differences between groups for BED_DI_ (p-value = 1×10^-10^), but not for BED_simp_ (p-value 0.9).

**Supplementary Figure 9.** Causal effect estimates of BED_DI_ (top two panels) and BED_simp_ (bottom two panels) from the CSF analyses. The boxplots show the distributions of restricted mean survival time (RMST) or survival probability (SP) causal effects over 10-fold cross validation folds on the training data.

| **Feature** | **RMST** | | | **SP** | | |
| --- | --- | --- | --- | --- | --- | --- |
|  | **Estimate** | **SE** | **p-value** | **Estimate** | **SE** | **p-value** |
|  | **Causal variable = BED_DD_** | | | | | |
| Intercept | 90.713 | 110.773 | 0.413 | 15.918 | 11.656 | 0.172 |
| Sex | 0.076 | 0.424 | 0.858 | -0.083 | 0.107 | 0.442 |
| Smoking_PY_ | -0.010 | 0.007 | 0.183 | -0.001 | 0.001 | 0.300 |
| Stage_numeric_ | -0.711 | 0.158 | 7.44×10^-6^ | -0.051 | 0.042 | 0.218 |
| HPV_Positive_ | 0.009 | 0.495 | 0.986 | 0.009 | 0.059 | 0.874 |
| HPV_Unknown_ | -0.075 | 0.504 | 0.881 | -0.007 | 0.079 | 0.930 |
| Chemo | 0.533 | 0.510 | 0.297 | 0.019 | 0.057 | 0.744 |
| RT_year_ | -0.044 | 0.055 | 0.422 | -0.008 | 0.006 | 0.177 |
| Age | 0.013 | 0.019 | 0.491 | 0.003 | 0.005 | 0.498 |
|  | **Causal variable = BED_DI_** | | | | | |
| Intercept | 154.661 | 152.101 | 0.309 | 20.802 | 20.188 | 0.303 |
| Sex | 0.014 | 0.578 | 0.981 | -0.143 | 0.156 | 0.359 |
| Smoking_PY_ | -0.011 | 0.011 | 0.308 | -0.001 | 0.002 | 0.463 |
| Stage_numeric_ | -0.585 | 0.259 | 0.024 | -0.039 | 0.062 | 0.535 |
| HPV_Positive_ | -0.435 | 0.705 | 0.538 | -0.040 | 0.091 | 0.655 |
| HPV_Unknown_ | -0.552 | 0.671 | 0.411 | -0.040 | 0.118 | 0.735 |
| Chemo | 0.449 | 0.738 | 0.543 | 0.025 | 0.101 | 0.803 |
| RT_year_ | -0.076 | 0.076 | 0.317 | -0.010 | 0.010 | 0.306 |
| Age | 0.013 | 0.026 | 0.618 | 0.005 | 0.007 | 0.508 |

**Supplementary Table 2.** Best linear projections for CSF models that used either BED_DD_ or BED_DI_ as the causal variable, which indicate how each covariate feature influenced the CSF causal effect estimates for the RMST (restricted mean survival time) and survival probability (SP) metrics. RMST and SP here were calculated 10 and 8 years after treatment, respectively. These time points were chosen because RMST and SP were maximal there. SE represent cluster- and heteroskedasticity-robust standard errors.

**References**

1 Welch ML, et al. in The Cancer Imaging Archive The Cancer Imaging Archive (ed The Cancer Imaging Archive) (The Cancer Imaging Archive, 2023).

2 Kazmierski M, et al. Multi-institutional prognostic modeling in head and neck cancer: evaluating impact and generalizability of deep learning and radiomics. Cancer Res Commun (2023) 3:1140-1151. doi: 10.1158/2767-9764.CRC-22-0152

3 Fowler JF. 21 years of biologically effective dose. Br J Radiol (2010) 83:554-568. doi: 10.1259/bjr/31372149

4 Withers HR, Taylor JM, Maciejewski B. The hazard of accelerated tumor clonogen repopulation during radiotherapy. Acta Oncol (1988) 27:131-146. doi: 10.3109/02841868809090333

5 Shuryak I, Hall EJ, Brenner DJ. Dose dependence of accelerated repopulation in head and neck cancer: supporting evidence and clinical implications. Radiother Oncol (2018) 127(1):20-6. doi: 10.1016/j.radonc.2018.02.015

6 Breiman L. Random forests. Mach Learn (2001) 45:5-32. doi: 10.1023/a:1010933404324

7 Lundberg SM, Lee SI. A Unified Approach to Interpreting Model Predictions. arXiv (2017) 1705:07874. doi: 10.48550/arXiv.1705.07874

8 Package ‘grf’ (2022). https://cran.r-project.org/web/packages/grf/index.html

9 Zhang Y, Li H, Ren G. Estimating heterogeneous treatment effects in road safety analysis using generalized random forests. Accid Anal Prev (2022) 165:106507. doi: 10.1016/j.aap.2021.106507

10 Athey S, Wager S. Estimating Treatment Effects with Causal Forests: An Application. Observational Studies (2019) 5:37-51. doi: 10.48550/arXiv.1902.07409

11 Bodory H, Busshoff H, Lechner M. High resolution treatment effects estimation: uncovering effect heterogeneities with the modified causal forest. Entropy (Basel) (2022) 24. doi: 10.3390/e24081039

12 Bonander C, Svensson M. Using causal forests to assess heterogeneity in cost-effectiveness analysis. Health Econ (2021) 30:1818-1832.
